# Report from the 29th Meeting on Toxinology, “Toxins: From the Wild to the Lab”, Organized by the French Society of Toxinology on 30 November–1 December 2023

**DOI:** 10.3390/toxins16030147

**Published:** 2024-03-13

**Authors:** Pascale Marchot, Ziad Fajloun, Christian Legros, Évelyne Benoit, Sylvie Diochot

**Affiliations:** 1 Laboratoire Architecture et Fonction des Macromolécules Biologiques (AFMB), CNRS/Aix-Marseille Université, Faculté des Sciences—Campus Luminy, F-13288 Marseille, CEDEX 09, France; pascale.marchot@univ-amu.fr; 2 Laboratory of Applied Biotechnology (LBA3B), AZM Center for Research in Biotechnology and Its Applications, Doctoral School for Sciences and Technology, Lebanese University, Tripoli 1300, Lebanon; ziad.fajloun@ul.edu.lb; 3 Department of Biology, Faculty of Sciences 3, Campus Michel Slayman Ras Maska, Lebanese University, Tripoli 1352, Lebanon; 4 Univ. Angers, INSERM, CNRS, MITOVASC, Equipe CarMe, SFR ICAT, F-49000 Angers, France; christian.legros@univ-angers.fr; 5 Service d’Ingénierie Moléculaire Pour la Santé (SIMoS), EMR CNRS/CEA 9004, Département Médicaments et Technologies Pour la Santé (DMTS), Institut des Sciences du Vivant Frédéric Joliot, Université Paris-Saclay, CEA, F-91191 Gif-sur-Yvette, France; 6 Institut de Pharmacologie Moléculaire et Cellulaire, Université Côte d’Azur, CNRS UMR7275, INSERM U1323, Sophia Antipolis, F-06560 Valbonne, France

**Keywords:** animal toxin, bacterial toxin, marine toxin, toxinomics, venomics, structure–function interactions, therapeutic drug

## Abstract

The French Society of Toxinology (SFET), which celebrated its 30th anniversary this year, organized its 29th annual Meeting (RT29), shared by 87 participants, on 30 November–1 December 2023. The RT29 main theme, “Toxins: From the Wild to the Lab”, focused on research in the field of animal venoms and animal, bacterial, fungal, or plant toxins, from their discovery in nature to their study in the laboratory. The exploration of the functions of toxins, their structures, their molecular or cellular ligands, their mode of action, and their potential therapeutic applications were emphasized during oral communications and posters through three sessions, of which each was dedicated to a secondary theme. A fourth, “miscellaneous” session allowed participants to present recent out-of-theme works. The abstracts of nine invited and 15 selected lectures, those of 24 posters, and the names of the Best Oral Communication and Best Poster awardees, are presented in this report.

## 1. Acknowledgements

**Figure 1:**
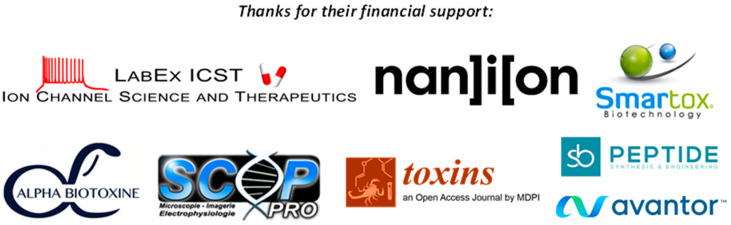
Sponsors’ logos.

We warmly acknowledge the contribution of all the people who work daily at ensuring the national and international shinning of the French Society of Toxinology (SFET, http://sfet.asso.fr/international, accessed since 1 September 2023). We are also grateful to those who made the 29th Meeting on Toxinology (RT29) and commemoration of the SFET 30th anniversary, held at the Institut Pasteur of Paris on 30 November–1 December 2023, a success, with special thanks to our always faithful or new sponsors (Figure 1).

## 2. Preface

The central theme of the SFET RT29, “Toxins: From the Wild to the Lab”, focused on research in the field of animal venoms and animal, bacterial, fungal or plant toxins, from their discovery in Nature to their study in the laboratory. The exploration of the functions of toxins, their structures, their molecular or cellular ligands, their mode of action, and their potential therapeutic applications were emphasized during oral communications and posters through three sessions dedicated to “Venom Research”, “Toxin functional exploration” and “Toxin-based therapeutics”, respectively. Recent progress within these three themes was presented by ten invited experts in the field, originating from six countries (Belgium, France, Germany, Ireland, Portugal and the United States of America).

Fifteen other speakers, selected on abstract among researchers, postdoctoral fellows and students, also presented their work in these sessions or in the fourth, “Miscellaneous” session traditionally dedicated to recent, albeit out-of-theme studies. Twenty-seven posters were displayed, presented and discussed all throughout the meeting, of which 15 were also selected for very dynamic, 2 min flash-talks. Noteworthy, 38% of the 87 participants of the RT29 were foreigners, a ratio that, once again, highlights the international attractiveness of the Meetings on Toxinology held by the SFET.

The rich complexity of toxins that compose animal venoms can be explored through the extraordinary variety of venomous animal species, including not only those spectacular species found in Africa, America, Asia and Oceania, but also those more discrete species that European people can meet in their nearby environment. Hence, *venom research in the wild* was well-illustrated by reports on the physiological, ecological, and evolutionary factors that drive composition and efficacy of European spider venoms, and by the chemical and functional ecological approach performed on 15 Amazonian ant species.

In turn, bringing venoms *from the wild to the lab* must be rigorously supervised by experts able to identify species accurately, while respecting a regulatory environment with ever-growing requirements and complexity, for keeping animals in captivity and ensuring their well-being. The many and various innovative techniques currently used in lab to study the composition of venoms were highlighted mainly on snake venoms, albeit examples related to cone snail, spider and honeybee venoms were also provided. For example, we learned how organoids in culture can be used to assay the effects of snake venoms on haemostasis, and that venoms of marine cones and ants combine multiple original peptides targeting an increasing number of previously “orphaned” receptors.

*Toxin functional exploration in the lab* was nicely pictured by reports on novel molecular targets and/or mechanisms of action of a full “palette” of toxins from bacterial, dinoflagellate, cone, honeybee, ant, scorpion and snake origins.

Academic and industrial researchers are always ready to harness their originality to develop new diagnostic or therapeutic tools. *Toxin-based therapeutics* opportunities and strategies were exemplified by reports on caged natural peptides for spatiotemporal control of neuronal and cardiac ion channels; on the recent progress in protein engineering to create novel cancer biomarkers or antivenoms; on the possibilities and pitfalls of peptides to treat lung disease; on the multiple therapeutic applications of *Botulinum* neurotoxin; on inhibition, by honeybee toxins, of the early steps of Toscana virus replication; on snake toxin-based drugs and drugs against snake toxins; and on the involvement of a mammalian, genuine ortholog of β-neurotoxic sPLA2 from snake venom in Alzheimer’s disease.

Finally, the *miscellaneous* session described recent advances in the detection or capture of tetrodotoxins; in the structural diversity, pharmacological relevance and synthesis strategies of conotoxins; and in assembly of ant genome at the chromosome-level.

**Figure 2:**
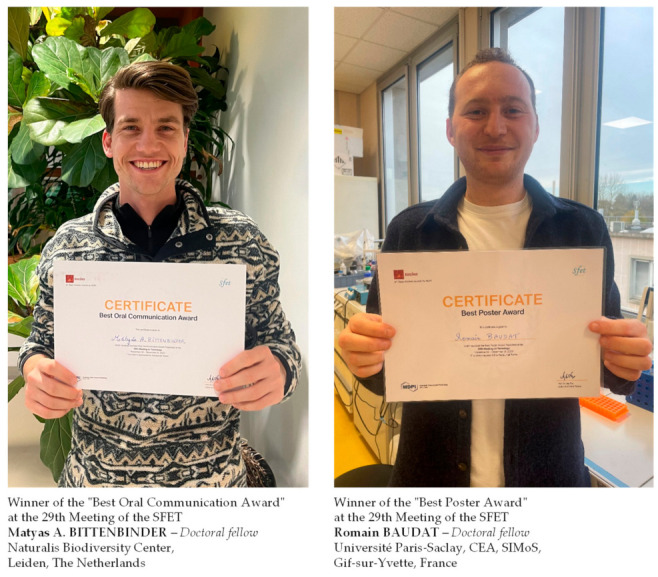
The “Best Oral Communication” and “Best Poster” Awardees at the 29th Meeting on Toxinology of the French Society of Toxinology (SFET).

Owing to a donation from the MDPI journal Toxins, two awards of EUR 200 each were given to the best oral communication and best poster (Figure 2), both selected via a vote by a jury comprising the invited speakers and of the members of the SFET Board of Directors.

And above all, we warmly thank the Editors of Toxins for supporting the publication of a Special Issue also entitled “Toxins: From the Wild to the Lab” and that will bring together this meeting report and peer-reviewed original articles and reviews. We are confident that this Special Issue will be attractive to all, including those of our colleagues who could not attend the RT29 meeting, and that it will represent a comprehensive source of information for researchers and students in toxinology.

## 3. Scientific and Organizing Committee (SFET Board of Directors)

Évelyne Benoit, CEA de Saclay, Gif-sur-Yvette, France

Katrina Campbell, Institute for Global Food Security, Queen’s University Belfast, Belfast, Northern Ireland

Alexandre Chenal, Institut Pasteur, Paris, France

Sylvie Diochot, Institut de Pharmacologie Moléculaire et Cellulaire, Valbonne, France

Sébastien Dutertre, Institut des Biomolécules Max Mousseron, Montpellier, France

Ziad Fajloun, Doctoral School of Science and Technology, Lebanese University, Tripoli, Lebanon

Daniel Ladant, Institut Pasteur, Paris, France

Christian Legros, Université d’Angers, Angers, France

Pascale Marchot, CNRS/Aix-Marseille Université, Marseille, France

Michel R. Popoff, retired from Institut Pasteur, Paris, France

Loïc Quinton, Université de Liège, Liège, Belgium

Michel Ronjat, retired from Institut du thorax, Université de Nantes, Nantes, France

## 4. Invited Lectures (When More Than One Author, the Underlined Name Is That of the Presenter)

### 4.1. Venom Potency and Composition of European Spiders

Michel M. Dugon *
Venom Systems and Proteomics Lab, School of Natural Sciences, Ryan Institute, University of Galway, H91 Galway, Ireland.
* Correspondence: michel.dugon@nuigalway.ie

**Abstract:** Venom studies have traditionally focused on large or medically significant organisms, such as snakes, lizards, scorpions and tarantulas. This has been performed either for commodity, i.e., the ability to extract large amounts of venom from a single specimen, or because the scientific community is fixated on deadly organisms. This approach, although understandable in many aspects, overlooks the vast majority of venomous organisms, some of which live (literally) on our doorstep. Spiders form the most diverse clade of terrestrial venomous organisms, with over 50,000 species described so far. Globally, spiders envenomate and consume 400 to 800 million tons of arthropods every year and may produce more than 10 million peptides with potential applications. However, most spiders are relatively small and of negligible medical importance. As a result, their venom remains poorly studied, with only a fraction of all species ever investigated. To truly appreciate the 4500+ species of European spiders and understand the potential role of their venom as a source of compounds for biomedical and agricultural applications, we need to shift the current paradigm where measures of venom efficiency focus on median Lethal Dose (LD50), and instead investigate the physiological, ecological and evolutionary factors that drive venom composition and efficacy.

**Keywords:** ecology; evolution; spider; venom

### 4.2. Exploring the Diversity of Amazonian Ants to Discover Novel Neurotoxic Venom Peptides

Axel Touchard ^1,2,^*

 ^1^ CNRS, UMR Ecologie des forêts de Guyane, 97304 Kourou, France.
 ^2^ Department of entomology, Cornell University, 14853 Ithaca, NY, USA.
 * Correspondence: axel.touchard2@gmail.com

**Abstract:** Ants, which dominate most terrestrial environments, are one of the most abundant groups of venomous organisms on Earth and are among of the leading predators of invertebrates in most ecosystems. To subdue their prey, they have evolved an arsenal of adaptations and weapons, including potent venoms with paralytic and lethal effects on many types of arthropods. So far, ant venoms received little attention yet hold great promise for the discovery of novel insecticidal peptides. In this talk, I will present the ant venom research conducted in the rainforest of the French Guiana with a focus on the unveiling of Cysteine-Rich Poneritoxins (CRPs) from *Anochetus emarginatus* venom, a novel class of neurotoxins. To gain further insights into the evolution of CRPs, a chemical and functional ecological approach have been performed on 15 Amazonian ant species with contrasting ecological traits. Proteo-transcriptomic analyses revealed markedly different venom peptide compositions among the 15 species, but CRPs are closely related to other peptides with a single disulphide bond identified as minor toxins from the venoms of the genus *Odontomachus*. The ancestral state reconstruction of CRP peptides by using similarity-based searches in the genome of 163 ant species showed a recent appearance of this toxin family suggestive of a rapid diversification in a restricted clade of Formicidae. Then, the analysis of venom bioactivities (insect paralysis, lethality, cytotoxicity, and vertebrate pain) conducted among the 15 species suggests that CRP peptides evolved under an exclusively offensive purpose to incapacitate invertebrate prey. Further paralytic assays in bees and blowflies with isolated synthetic CRPs showed that venom CRPs exhibit taxonomic specificity among insect prey. Altogether, these results emphasize that ant venoms have evolved promising insecticidal neurotoxins that could be exploited to design more effective and pest-specific bioinsecticides.

**Keywords:** ant; insect prey; neurotoxin; venom

### 4.3. How Alphabiotoxine Brings the Wild to Your Lab

 Aude Violette *, Rudy Fourmy
 Alphabiotoxine Laboratory, 7911 Montroeul-au-Bois, Belgium.
 * Correspondence: aude.violette@alphabiotoxine.com

**Abstract:** Today, working on research and development projects related to venoms while respecting a regulatory environment with ever growing requirements and complexity has become a real challenge. From animal sourcing to venom collection, going through animal husbandry, the constraints are numerous. Despite being cumbersome, these regulations are necessary to ensure an ethical production of venoms, in the respect of animal species protection and animal well-being. The scientific quality requirements meet the regulatory constraints in the need for traceability. From the wild to the lab, the study of toxins is a long road.

**Keywords:** Alphabiotoxine; venom production; venomous animal milking

### 4.4. The CNF1 Toxin Enhances the capacity of Extraintestinal Pathogenic Escherichia coli to Compete for Gut Colonization

 Emmanuel Lemichez *, Serena Petracchini, Landry Tsoumtsa Meda, Elea Paillares, Marie Anne Nahori, Amel Mettouchi
 Unité des Toxines Bactériennes, Département de Microbiologie, Institut Pasteur, CNRS UMR6047, INSERM U1306, Paris, France.
 * Correspondence: emmanuel.lemichez@pasteur.fr

**Abstract:** The Cytotoxic Necrotizing Factor 1 (CNF1) is a protein toxin produced by pathogenic strains of *Escherichia coli* that catalyses the deamidation of a glutamine residue of Rho GTPases thereby strengthening integrin signalling that in turn ensures an efficient invasion of host epithelial cells by *E. coli* expressing FimH adhesin of type 1 pili. The function of this toxin in infection and population dynamics of *E. coli* has long been unsolved. Recent bioinformatics analyses of a large collection of *E. coli* genomes from EnteroBase, enriched in clinical isolates of worldwide origins, suggest the CNF1 toxin encoding gene, *cnf1*, is preferentially distributed in four common sequence types (ST) encompassing the pandemic *E. coli* MDR lineage ST131. This lineage is responsible for a majority of extraintestinal infections that escape first-line antibiotic treatment, with known enhanced capacities to colonize the gastrointestinal tract. Statistical projections based on this dataset point to a global expansion of *cnf1*-positive multidrug-resistant ST131 strains from subclade *H*30Rx/C2, accounting for a rising prevalence of *cnf1*-positive strains in 131 sequence type. Despite the absence of phylogeographical signals, *cnf1*-positive isolates segregated into clusters in the ST131-*H*30Rx/C2 phylogeny, sharing a similar profile of virulence factors and the same *cnf1* allele. I will discuss recent unpublished data suggesting that the dominant expansion of *cnf1*-positive strains in ST131-*H*30Rx/C2 is likely related to the competitive advantage conferred by *cnf1* between ExPEC strains for gut colonization.

**Keywords:** CNF1; colonization; ExPEC; gut; Rho GTPase

### 4.5. Characterization of α-Conotoxins and Their Structure Activity Relationships by Two-Electrode Voltage Clamp Analysis

 Annette Nicke *
 Walther Straub Institute of Pharmacology and Toxicology, Faculty of Medicine, LMU Munich, 80336 Munich, Germany.
 * Correspondence: annette.nicke@lrz.uni-muenchen.de

**Abstract:** Nicotinic acetylcholine receptors (nAChRs) represent a complex family of pentameric ion channels formed by the variable assembly of homologous subunits. Their prominent functions in neuro- and neuromuscular transmission are reflected by a variety of natural defence mechanisms and prey capture strategies which target this receptor and make them important targets for drug development. α-Conotoxins, small peptides isolated from predatory marine snails, are the most selective ligands at nAChRs and represent invaluable tools to investigate their physiological and molecular functions. While more or less potent and subtype selective α-conotoxins have been identified for most nAChR subtypes, α4-containing receptors appear to be largely resistant against inhibition by α-conotoxins. Together with the increasing availability of high-resolution structures of nAChRs and new modelling approaches this provides versatile models to study protein peptide interactions. In our lab we use the *Xenopus laevis* oocyte expression system and two-electrode voltage-clamp analysis to investigate structure function relations of native and mutant α-conotoxins. Here, we will present our recording system, recent approaches to investigate determinants of subtype selectivity and test the hypothesis that the α4β2 subtype has unique structural features that restrict conotoxin access to the ligand-binding site.

**Keywords:** conotoxin; nAChR nicotinic acetylcholine receptor; two-electrode voltage-clamp

### 4.6. The Sobering Sting: A Novel Cone Snail Venom-Derived Antagonist of Cannabinoid Receptors

 Dongchen An ^1^, Guilherme Salgado Carrazoni ^2^, Ben-Hur Souto Das Neves ^2^, Rudi D’Hooge ^2^, Jan Tytgat ^1^, Steve Peigneur ^1,^*
 ^1^ Toxicology and Pharmacology, University of Leuven (KU Leuven), Campus Gasthuisberg, O &N2, PO. Box 922, 3000 Leuven, Belgium.
 ^2^ Laboratory of Biological Psychology and Leuven Brain Institute, Faculty of Psychology and Educational Sciences, KU Leuven, Tiensestraat 102, 3000 Leuven, Belgium.
 * Correspondence: steve.peigneur@kuleuven.be

**Abstract:** Cannabinoid receptors (CB1 and CB2) are promising targets for diverse diseases, such as pain, inflammation, and neurological diseases. However, only a few ligands of CB receptors have reached clinical application so far. In the present study, we focused on the venom of *Stephanoconus* snails and found an endocannabinoid-like molecule, oleoyl serotonin (OS), active on CB1 and CB2. To determine the effects of OS, CB1 and CB2 receptors were functionally expressed in *Xenopus laevis* oocytes, together with the G protein-coupled inwardly rectifier potassium channels (GIRK1/2), and a regulator of G protein signalling (RGS4). Ion currents were measured with a two-electrode voltage-clamp system. Results show that OS competitively blocks GIRK1/2 currents induced by WIN55,212-2 (WIN), a non-selective CB agonist, in the CB1-GIRK1/2-RGS4 system. Interestingly, OS acts as a competitive antagonist of CB1 receptors and as a non-competitive blocker of CB2 receptors. To the best of our knowledge, OS is the first venom-derived ligand modulating CB receptors. Furthermore, OS does not show significant activity on serotonin, histamine, opioid and nicotinic receptors, TRP channels or voltage-gated sodium, potassium or calcium channels. To investigate the critical domains of the CB receptor for OS activity, we constructed a chimeric CB1/2 receptor by replacing the transmembrane domain V (TMV)-intracellular loop 3 (ICL3)-TMVI domain (TMV-ICL3-TMVI) of the CB2 receptor with that of the CB1 receptor. Structure-function studies revealed that the TMV-ICL3-TMVI domain of the CB2 receptor is essential for OS to exert its antagonistic effect on CB2 receptors. Furthermore, in vivo evaluation of OS on brain-related neurological functions in mice suggests that OS has an anxiolytic effect, diminishing the stress response in stressful situations. This study highlights the potential of animal venom components as a promising library for the discovery of novel CB receptor ligands.

**Keywords:** antagonist; cannabinoid receptor; cone snail venom; learning and memory deficit

### 4.7. The Multiple Therapeutic Applications of Botulinum Neurotoxin: The Same Mechanism at Different Nerve Endings

 Bernard Poulain ^1,^*, Michel R. Popoff ^2^
 ^1^ Institut des Neurosciences Cellulaires et Intégratives, UPR3212 CNRS, 67084 Strasbourg, France.
 ^2^ Unité Toxines Bactériennes, Institut Pasteur, 75015 Paris, France.
 * Correspondence: Bernard.Poulain@cnrs.fr

**Abstract:** Botulinum neurotoxins (BoNT, ~150 kDa) are bacterial proteins that inhibit SNARE-mediated exocytosis. When administered peripherally, they do not cross the blood-brain barrier and inhibit the release of neurotransmitters (mainly acetylcholine, ACh) in striated muscles and glands. The muscle-relaxing effects of BoNT began to be exploited over 40 years ago, first in ophthalmology, then in neurology, physical and rehabilitation medicine (movement disorders, spasticity) and neuro-urology. BoNT is also used to treat hypersecretory glands. In aesthetics, toxin type A is used to erase wrinkles. It is also used to relieve pain (certain indications). The medical indications for botulinum toxin are so numerous and varied that they raise the question of the underlying mechanisms (single or multiple?) and the identity of the cell type(s) targeted by BoNT. In the context of medical use (low concentrations of BoNT), an effect on non-neuronal cells has not yet been demonstrated. In fact, neurons are the only cell type meeting the criteria that define very high sensitivity to BoNT. These are (i) membrane expression of appropriate co-receptors for the type of BoNT used (a vesicular protein together with a tri- or di-sialo-ganglioside) to enable very high affinity binding; (ii) the presence of strong endocytotic activity (recycling of synaptic vesicles or other endocytosis pathway) to allow efficient internalisation of BoNT; (iii) sorting of captured BoNT molecules into an acidifying intracellular compartment to allow translocation of the catalytic domain into the cytosol, and iv) expression of SNARE isoforms cleavable by the catalytic domain of BoNT. Variations in the expression of protein receptor isoforms between neurons explain why different types of BoNT produce distinct effects. In addition, depending on the nerve terminals they enter, their intracellular routing can differ leading to different manifestations. For instance, when administered close to free sensory nerve endings, a significant fraction of BoNT molecules undergoes retroaxonal ascent towards the nuclei containing neuronal cell bodies. There, BoNT blocks SNARE-mediated exocytosis (e.g., leading to inhibition of glutamate and peptide release and incorporation of receptors into the membrane). Pain relief induced by injection of BoNT in the periphery most likely involves this type of action in dorsal root ganglia or trigeminal ganglia. In conclusion, although the spectrum of medical indications benefiting from BoNT injection is very broad, in all cases, inhibition of BoNT-induced exocytosis mechanisms in neurons provides the desired therapeutic gain.

**Keywords:** botulinum neurotoxin; botulino-therapy; medical indication

### 4.8. Caged Natural Peptides for the Spatio-Temporal Control of Neuronal and Cardiac Ion Channels

 Michel De Waard *
 L’institut du thorax, Inserm UMR 1087/CNRS UMR 6291, 44000 Nantes, France.
 * Correspondence: michel.dewaard@univ-nantes.fr

**Abstract:** For this topic, I will present data on a versatile technology that enables natural peptide caging, a process whereby the peptide pharmacophore is neutralized by a photosensitive protecting group. This technology provides full control over the pharmacological action of a peptide and allows one to spatially and temporally restrict its activity to a given tissue in vivo. In spite of the fact that UV light is not the most adequate for tissue penetration, we managed to develop several applications in vivo whereby toxin caging and light-induced uncaging provide a selective muscle paralysis, a targeted control of heart rhythm or a block of action potential generation in a neuronal sub-compartment. We shall also explain what to expect in the future for this technology and how it may be envisioned for clinical applications.

**Keywords:** caged peptide; photopharmacology; spatio-temporal control

### 4.9. Snake Venom: Toxin-Based Drugs and Drugs for Toxins

 Pedro Alexandrino Fernandes *
 LAQV-REQUIMTE/Department of Chemistry and Biochemistry, Faculty of Sciences, University of Porto, Porto 4169-007, Portugal.
 * Correspondence: pafernan@fc.up.pt

**Abstract:** The snake has been feared since ancient times, partly due to the devastating power of its venom. However, many mythologies across the globe also attribute healing powers to the snake, often elevating it to a divine status. This lecture addresses both facets of the snake—the dangerous animal responsible for snakebite envenoming, the deadliest of all neglected diseases, and the animal that holds a potential cure for some diseases of great societal importance in its venom. We will review the chemical diversity of snake venom [1], address modern studies that try to clarify the mechanism of action of some major venom toxins such as snake venom phospholipases A2 and metalloproteinases [2,3], and explain how to harness the bioactive power of venoms to develop new drugs, illustrating some examples under development in our lab.

 
**References**
1. Oliveira, A.L.; Viegas, M.F.; da Silva, S.L.; Soares, A.M.; Ramos, M.J.; Fernandes, P.A. The chemistry of snake venom and its medicinal potential. *Nat. Rev. Chem.* **2022**, *6*, 451–469.
2. Castro-Amorim, J.; Novo de Oliveira, A.; Da Silva, S.L.; Soares, A.M.; Mukherjee, A.K.; Ramos, M.J.; Fernandes, P.A. Catalytically active snake venom PLA2 enzymes: an overview of its elusive mechanisms of reaction. *J. Med. Chem.* **2023**, *66*, 5364–5376.
3. Castro-Amorim, J.; Oliveira, A.; Mukherjee, AK.; Ramos, M.J.; Fernandes, P.A. Unraveling the reaction mechanism of Russell's viper venom factor X activator: a paradigm for the reactivity of zinc metalloproteinases? *J. Chem. Inf. Model* **2023**, *63*, 4056–4069.

**Keywords:** drug discovery; snake venom; toxin enzyme

## 5. Oral Presentations (When More Than One Author, the Underlined Name Is That of the Presenter)

### 5.1. Acetylcholine Binding Protein Affinity Profiling of Neurotoxins in Snake Venoms with Parallel Toxin Identification by High Throughput Venomics

 Giulia Palermo ^1,2^, Wietse Schouten ^1,2^, Luis L. Alonso ^1,2^, Jeroen Kool ^1,2^, Julien Slagboom ^1,2,^*
 ^1^ Amsterdam Institute of Molecular and Life Sciences, Division of BioAnalytical Chemistry, Department of Chemistry and Pharmaceutical Sciences, Faculty of Science, Vrije Universiteit Amsterdam, Amsterdam 1081HV, The Netherlands.
 ^2^ Centre for Analytical Sciences Amsterdam (CASA), 1012 WX Amsterdam, The Netherlands.
 * Correspondence: j.slagboom@vu.nl

**Abstract:** Snakebite is a concerning issue and is considered a Neglected Tropical Disease. Three-finger toxins (3FTxs) found in snake venoms are the main responsible toxins for neurotoxic effects since they have high affinities for nicotinic acetylcholine receptors (nAChRs) as well as for other receptor types. Due to their small molecular size, 3FTxs are only weakly immunogenic and therefore during immunization for antivenom production, 3FTxs are often not appropriately targeted. Alternative snakebite treatment approaches for neutralizing 3FTx are therefore interesting. This study aims at presenting and applying an analytical method for investigating the therapeutic potential of the acetylcholine binding protein (AChBP), an efficient nAChR mimic that is able to capture 3FTxs, for treatment of elapid snakebites. In the here presented analytical methodology, snake venom toxins in crude venoms were firstly separated and characterised by accurate mass assessment by means of high-performance liquid chromatography coupled to mass spectrometry (HPLC-MS) with parallel toxin identification using high throughput venomics. By subsequent nanofractionation analytics, binding profiling of toxins to AChBP was achieved with a post-column plate reader-based fluorescence-enhancement ligand displacement bioassay. The integrated method was established and then applied to profiling venoms of nine snakes from the Elapidae family (*Naja kaouthia*, *N. mossambica*, *N. naja*, *N. haje*, *Ophiophagus hannah*, *Bungarus multicinctus*, *B. caeruleus*, *B. candidus* and *Dendroaspis polylepis*). The methodology demonstrated that AChBP is able to effectively bind long-chain 3FTxs with relatively high affinity but has low or no binding affinity towards short-chain 3FTxs and as such provides an efficient analytical platform to investigate binding affinities of 3FTxs to AChBP and mutants thereof, and to rapidly identify the bound toxins.

**Keywords:** AChBP; neurotoxicity; proteomics; snake venom; three-finger toxin

### 5.2. Bloody Insights: Using Organ-on-Chip Technology to Study Haemorrhagic Activities of Snake Venoms on Endothelial Tubules

 Mátyás A. Bittenbinder ^1,2,3,#^, Flavio Bonanini ^4,#^, Dorota Kurek ^4^, Jeroen Kool ^2,3,$,^*, Freek J. Vonk ^1,2,3,$,^*
 ^1^ Naturalis Biodiversity Center, 2333 CR Leiden, The Netherlands.
 ^2^ Amsterdam Institute of Molecular and Life Sciences, Division of BioAnalytical Chemistry, Vrije Universiteit Amsterdam, 1081 HV Amsterdam, The Netherlands.
 ^3^ Centre for Analytical Sciences Amsterdam (CASA), 1081 HV Amsterdam, The Netherlands.
 ^4^ Mimetas, 2333 CR Leiden, The Netherlands.
 ^#^ Equally contributing first authors.
 ^$^ Equally contributing senior authors.
 * Correspondence: j.kool@vu.nl

**Abstract:** Snakebite envenomation is a major public health issue, which causes severe morbidity and mortality affecting millions of people annually. Studying the haemorrhagic effects in vitro has been challenging as the effects observed in vivo (i.e., rapid onset of haemorrhagic activities) is not observed during in vitro studies. To date, the study of tissue-damaging effects of snake venom toxins has been largely based on two-dimensional cell culture models. These do not have the tubular morphology of vasculature found in vivo and lack important environmental cues from the cellular microenvironment, such as interaction with the extracellular matrix (ECM) and exposure to flow. To bridge this ‘discrepancy’ in the tissue-damaging effects observed in vitro and in vivo models, we implemented organ-on-a-chip technology to investigate the effects of four different snake venoms on a microfluidic blood vessel model. We were able to multiplex several readouts to comprehensively assess the tissue-damaging activities of a panel of snake venoms on a human blood vessel model grown in an organ-on-chip platform. We adapted image-based protocols using a fluorescence microscope plate-reader to assess endothelial barrier function for monitoring the vascular leakage kinetically after venom exposure. Simultaneously, we assessed morphology and viability allowing us to differentiate between different modes of toxicity of the different venoms, such as blood vessel disruption without significant cell killing or extensive cell death without apparent structural disruption. Using this approach, we were able to differentiate between at least two distinct mechanisms by which the microvasculature is being affected. The first mechanism being delamination of the endothelial cell monolayer from its surroundings, the second mechanism involving disruption of the endothelial cell membrane.

**Keywords:** envenoming; haemorrhage; microfluidics; organ-on-chip; snakebite; tissue damaging activity; 3D cell culture

### 5.3. Characterization of the Venom of Conus Canonicus from Mayotte Island

 Zahrmina Ratibou ^1,^*, Sébastien Dutertre ^2^, Nicolas Inguimbert ^1^
 ^1^ CRIOBE, UAR CNRS-EPHE-UPVD 3278, Université de Perpignan Via Domitia, 58 avenue Paul Alduy, 66860 Perpignan, France.
 ^2^ IBMM, Université Montpellier, CNRS, ENSCM, 34093 Montpellier, France.
 * Correspondence: zahrmina.ratibou@univ-perp.fr

**Abstract:** Cone snails are carnivorous marine predators that prey on molluscs, worms, or fish. They are constituted with a venom gland that produces a highly diversified venom, mainly composed of small cysteine-rich peptides called conotoxins. They produce and purposefully inject different types of venom, whether they prey or defend themselves against predators. In this study, we focus on the venom of one molluscivorous cone, known as *Conus canonicus*, collected in the Mayotte islands, in the Indian Ocean. Transcriptomics studies enabled identification of 99 conotoxin sequences from 21 gene superfamilies, with the most expressed sequences belonging to the O, M, conkunitzin and T superfamilies. The composition of the predatory venom will be further investigated and compared with the distal, central, and proximal sections of the venom duct to reveal its origins. These results further expand our understanding of venom-ecology relationships in cone snails and provide a novel resource for potential drug discovery programs. This study takes part in my thesis work, which aims at characterizing and comparing cone snail venoms from Mayotte and French Polynesia.

**Keywords:** conotoxin; *Conus canonicus*; transcriptomics

### 5.4. Building Up a Pipeline for Assessing Potential Molecular Targets of Conotoxins with Unknown Mode of Action

 Juan Carlos García Galindo ^1,^*, Antonio Caballero Foncubierta ^1^, Sergio Chulián-Mantel ^2^, Manuel Jiménez Tenorio ^3^
 ^1^ Departamento de Química Orgánica-INBIO, Facultad de Ciencias, Universidad de Cádiz, 11510 Puerto Real, Spain.
 ^2^ Instituto de Biomoléculas-INBIO, Facultad de Ciencias, Universidad de Cádiz, 11510 Puerto Real, Spain.
 ^3^ Departamento de CMIM y Química Inorgánica-INBIO, Facultad de Ciencias, Universidad de Cádiz, 11510 Puerto Real, Spain.
 * Correspondence: juancarlos.galindo@uca.es

**Abstract:** Venomics studies on cone snails have unveiled a vast diversity of peptide neurotoxin structures present in their venom, named “conotoxins”. Conotoxins are the major constituents of the venom and target neural and neuromuscular receptors allowing the cone snail to paralyse or stun the prey. During the last two decades, the advances in transcriptomic techniques have let the discovery of more than 25,000 conotoxins. However, transcriptomic analysis does not permit to isolate the conotoxins and determine their molecular target and mode of action through bioassays. As a consequence, the number of conotoxins with a known mode of action is significantly lower: to date (September 2023) 999 conotoxins are registered in UNIPROT with a known molecular target. On the other hand, the use of AI advances in the study of the structure of proteins has come up with the development of powerful tools to model in silico their 3D structure. Among them, AlphaFold has revealed itself as a good approach to model protein structures. The comparison between the modelled structures and the experimental ones shows that the AlphaFold-predicted models and the experimentally solved structures contained in UNIPROT are alike in most of the cases. The bioactivity of conotoxins, and of any other peptidic signals, depends on their 3D structure that determines the assembly between the toxin and the target, but also on the sequence, on which it depends the chemical interactions within the pocket. In this approach, we will focus on the first factor: the 3D structure. We built a database with more than 25,000 published conotoxin sequences. By using AlphaFold, we will model their 3D structures in order to build another database containing their theoretical structures. Through the use of the recently disclosed bioinformatics tools RUPEE and Foldseek, we will compare the 3D structure of the published conotoxins with an unknown target with the structures of those with a known target obtained from UNIPROT. For those with an acceptable match, it is proposed that both conotoxins will likely have the same molecular target/mode of action. Even though the results will be biased by the number and types of known modes of action, data-mining will allow us to categorize conotoxins and lead future bioassay studies, allowing to optimize time and lab resources. Also, the result of those bioassays will help to test the model.

**Keywords:** AlphaFold; cone snail; conotoxin; Foldsee; mode of action; RUPEE; venomics

### 5.5. Cm39 (α-KTx 4.8): A Novel Scorpion Toxin That Inhibits Voltage-Gated K^+^ Channel Kv1.2 and Small- and Intermediate-Conductance Calcium-Activated K^+^ Channels KCa2.2 and KCa3.1

 Muhammad Umair Naseem ^1,^*, Georgina Gurrola-Briones ^2^, Margarita R. Romero-Imbachi ^3^, Jesús Angel Borrego Terrazas ^1^, Edson Carcamo-Noriega ^2^, José Beltrán-Vidal ^3^, Fernando Z. Zamudio ^2^, Kashmala Shakeel ^1^, Lourival D. Possani ^2^, Gyorgy Panyi ^1^
 ^1^ University of Debrecen, Faculty of Medicine, Department of Biophysics and Cell Biology, 4032 Debrecen, Hungary.
 ^2^ Universidad Nacional Autónoma de México, Departamento de Medicina Molecular y Bioprocesos, Mexico city 62210 Mexico.
 ^3^ Universidad del Cauca, Facultad de Ciencias Naturales, Departamento de Biología, Popayán 190002, Cauca, Colombia.
 * Correspondence: umairnaseem93@gmail.com

**Abstract:** A novel peptide toxin, Cm39, was identified in the venom of the Colombian scorpion *Centruroides margaritatus*. It is composed of 37 amino acid residues with a molecular weight of 3980.2 Da and folded by three disulphide bonds. The Cm39 sequence also contains the Lys-Tyr (KY) functional dyad required to block voltage-gated K^+^ (Kv1) channel. Amino acid sequence comparison with previously known K^+^ channel inhibitor scorpion toxins (KTx) and phylogenetic analysis revealed that Cm39 is a new member of α-KTx 4 family and registered with systematic number of α-KTx4.8. The full chemical synthesis and proper folding of Cm39 was obtained. The pharmacological properties of the synthetic peptide were determined using patch-clamp electrophysiology. Cm39 inhibited the voltage-gated K^+^ channel hKv1.2 with high affinity (Kd = 65 nM). The conductance-voltage relationship of Kv1.2 was not altered in the presence of Cm39, and the analysis of the toxin binding kinetics was consistent with a bimolecular interaction between the peptide and the channel; therefore, the pore blocking mechanism was proposed for the toxin-channel interaction. Cm39 also inhibited the Ca^2+^-activated KCa2.2 and KCa3.1 channels, with Kd = 575 nM, and Kd = 59 nM, respectively; however, the peptide did not inhibit hKv1.1, hKv1.3, hKv1.4, hKv1.5, hKv1.6, hKv11.1, mKCa1.1 potassium channels or the hNav1.5 and hNav1.4 sodium channels at 1 µM concentration. Understanding the unusual selectivity profile of Cm39 motivates further experiments to reveal novel interactions with the vestibule of toxin-sensitive channels.

**Keywords:** Kv1.2; KCa2.2; KCa3.1; patch-clamp electrophysiology; scorpion toxin

### 5.6. Secretion Signal of ExoY, a Nucleotidyl Cyclase of Pseudomonas Aeruginosa

 Jose Roberto Ponce-López, Vincent Deruelle, Dorothée Raoux-Barbot, Daniel Ladant, Undine Mechold *
 Unité de Biochimie des Interactions Macromoléculaires, Département de Biologie Structurale et Chimie, Institut Pasteur, CNRS UMR 3528, 75015 Paris, France.
 * Correspondence: undine.mechold@pasteur.fr

**Abstract:** The type III secretion system (T3SS) in *P. aeruginosa* is a needle complex that traverses 3 lipid membranes in order to selectively deliver the virulence effectors ExoS, ExoT, ExoU and ExoY into the infected host cell. This latter effector is a 42-kDa enzyme whose peculiarity is based on the production of the canonical cyclic nucleotides, cAMP and cGMP, as well as the non-canonical, cUMP and cCMP. To date, we know that ExoY requires F-actin found in the host cell to be activated. However, little is known about the mechanism of delivery of this toxin into the needle complex for its further secretion. The literature suggests that the composition in amino acid residues at the N-terminus of different effectors of the T3SS plays an important role for their secretion. We aim to understand the influence of the N-terminal amino acid composition of ExoY on its proper secretion into the infected cell. By mutagenesis, we found that the first 20 residues are important for the secretion of ExoY. The nucleotidyl cyclase activity of ExoY can be easily detected in supernatants of bacterial cultures induced to secrete the enzyme. We therefore suggest the use of ExoY as a reporter enzyme for the study of the secretion signal sequences of other toxins. In fact, replacing the first 20 residues of ExoY by those of other *P. aeruginosa* effectors (ExoS, ExoT and ExoU) enables secretion of the chimeras. We are currently investigating in how far the secretion and stability of ExoY depends on the presence of a chaperone. We expect that our contribution will help understand deeper the delivery mechanisms of the T3SS virulence effectors of *P. aeruginosa.*

**Keywords:** cNMP; ExoY; *Pseudomonas aeruginosa*; secretion; Type III secretion system (T3SS)

### 5.7. Activity of Snake Venom β-Neurotoxic sPLA2 Suggests How Its Mammalian Ortholog Is Involved in Alzheimer’s Disease

 Igor Križaj ^1,^*, Adrijan Ivanušec ^1,2^, Jernej Šribar ^1^, Adrijana Leonardi ^1^, Maja Zorović ^2^, Marko Živin ^2^, Peter Veranič ^2^
 ^1^ Jožef Stefan Institute, 1516 Ljubljana, Slovenia.
 ^2^ University of Ljubljana, Faculty of Medicine, 1000 Ljubljana, Slovenia.
 * Correspondence: igor.krizaj@ijs.si

**Abstract:** Snake venoms are a rich source of pharmacologically active secreted phospholipases A2 (sPLA2s), highly homologous to those found in mammals. Using the presynaptically neurotoxic (β-neurotoxic), group IIA sPLA2 from snake venom, GIIA, we described the main molecular steps in the process of β-neurotoxicity [1]. In a recent proof-of-concept study [2], we showed that the extracellularly applied mammalian GIIA affected nerve cells in a very similar manner to its β-neurotoxic snake venom relative, suggesting comparable molecular mechanisms. Since the elevated extracellular concentration of GIIA is one of the hallmarks of Alzheimer’s disease, we proposed an original molecular explanation for the onset of this pathology based on our results, which may offer new possibilities for treatment or diagnosis of this severe neurodegenerative disease.

 
**References**
1. Lomonte, B.; Križaj, I. *Handbook of Venoms and Toxins of Reptiles*. 2nd ed.; Stephen P. Mackessy, Ed.; 2021; CRC press Abingdon, Oxon OX14 4RN, pp. 389–411.
2. Ivanušec, A.; Šribar, J.; Veranič, P.; Križaj, I. Secreted phospholipases A2—Not just enzymes: revisited. *Int. J. Biol. Sci.* **2022**, *18*, 873–888.

**Keywords:** Alzheimer’s disease; β-neurotoxin; secreted phospholipase A2; snake venom

### 5.8. Recovery from the Neuroparalysis Induced by Neurotoxic Snake Venoms is Accelerated by an Agonist of the CXCR4 Receptor

 Marco Stazi ^1^, Samuele Negro ^1^, Giorgia D’Este ^1^, Andrea Mattarei ^2^, Aram Megighian ^1^, Michela Rigoni ^1^, Cesare Montecucco ^1,^*
 ^1^ Department of Biomedical Sciences, CNR Institute of Neuroscience, University of Padova, 35100 Padova, Italy.
 ^2^ Department of Pharmaceutical and Pharmacological Sciences, CNR Institute of Neuroscience, University of Padova, 35100 Padova, Italy.
 * Correspondence: cesare.montecucco@gmail.com

**Abstract:** Envenomation by snakes is a major neglected human disease even though it affects millions of people with sequelae and kills hundreds of thousands of them. A large toll is taken by snakes that inject neurotoxic venoms capable of inducing complete degeneration of motor axon terminals with rapid development of a peripheral neuroparalysis with severe respiratory deficit. The major venom component causing this degeneration are presynaptic acting PLA2s that hydrolyse phospholipids in lysophospholipids and fatty acids that induce large entry of Ca^2+^ into the neuronal cytosol. This leads to the activation of endogenous hydrolases that cause a complete degeneration of the motor axon terminals, which is followed by a regeneration program, only partially known in molecular terms. We recently identified two types of membrane receptors involved in the regeneration of the paralysed neuromuscular junction (NMJ). Here we report that this paralysis activates a proregenerative molecular axis, consisting of the CXCR4 receptor expressed at the stump of degenerated motor axon terminals and by the release of its ligand CXCL12α by surrounding Schwann cells. We also report that NUCC-390, a specific CXCR4 agonist acting like CXCL12 α, albeit with much better pharmacokinetics, shortens the time of recovery from neuroparalysis as determined by imaging, by different electrophysiological methods and by lung ventilation. NUCC-390 efficacy was tested in mice injected with venoms of three snake species of the *Bungarus* genus, and the venoms of the Papuan Taipan (*Oxyuranus scutellatus*), coral snake (*Micrurus nigrocinctus*) and Alpine viperas (*Vipera ammodytes* and *V. aspis*). In any case NUCC-390 was found to be very effective. This drug is non-toxic and does not cross the blood brain barrier. NUCC-390 meets all the requirements to become an additional therapeutic agent for many snake envenomings, which can be delivered after the bite to reduce the number of deaths by respiratory deficits and to shorten and improve functional recovery.

**Keywords:** neurotoxic venom; neurodegeneration; regeneration

### 5.9. The Venom of the Bee Apis Mellifera and Its Phospholipase A2 Inhibits the First Steps of Toscana Virus Replication

 Carole Yaacoub ^1,2,^*, Franck Touret ^1^, Ziad Fajloun ^2^, Bruno Coutard ^1^
 ^1^ Unité des Virus Emergents (UVE, Aix-Marseille Univ-IRD 190-Inserm 1207), 13000 Marseille, France.
 ^2^ Laboratory of Applied Biotechnology (LBA3B), Azm Center for Research in Biotechnology and its Applications, EDST, Lebanese University, 1300 Tripoli, Lebanon.
 * Correspondence: bruno.coutard@univ-amu.fr

**Abstract:** The sand flyborne Toscana virus (TOSV) poses an important health threat by causing human meningoencephalitis in the Mediterranean region during the summer months. Despite the severe impact of TOSV infections on human health, the absence of approved vaccines or effective antiviral treatments remains a pressing issue. This research delves into the exploration of the venom of the bee *Apis mellifera syriaca*, with a primary focus on its key constituents, melittin (MEL) and phospholipase A2 (PLA2), as potential antiviral agents against Toscana virus within Vero E6 cells. Our findings reveal the remarkable inhibitory potential of bee venom, with an EC50 value of 0.059 μg/mL, demonstrating its ability to combat Toscana virus effectively. Furthermore, our investigations highlight the important role of PLA2 in driving this potent antiviral activity. Interestingly, our experimental interventions at various stages of viral infection underscore PLA2 capacity to inhibit viral replication, whether introduced during viral adsorption or virus entry stages. These observations demonstrated the critical role of PLA2 in exerting its anti-Toscana virus at an early phase of infection. This study significantly advances our understanding of bee venom antiviral potential, with particular emphasis on the PLA2 component, offering promising insights into potential therapeutic strategies against Toscana virus infections, and probably against other Phleboviruses.

**Keywords:** antiviral effect; bee venom; Phlebovirus; phospholipase A2; Toscana virus

### 5.10. Ant Venom Peptides: From Screening Assays to Biological Targets Identification

 Guillaume Boy ^1^, Karthi Duraisamy ^2^, Elsa Bonnafé ^1^, Jérôme Leprince ^2^, Benjamin Lefranc ^2^, Michel Treilhou ^1^, Arnaud Billet ^1,^*
 ^1^ BTSB-EA 7417, Université de Toulouse, Institut National Universitaire Jean-François Champollion, Place de Verdun, 81012 Albi, France.
 ^2^ Inserm U1239, Différenciation et Communication Neuronale et Neuroendocrine, Normandie Univ, UNIROUEN, Plate-forme de Recherche en Imagerie Cellulaire Normandie (PRIMACEN), 76000 Rouen, France.
 * Correspondence: arnaud.billet@univ-jfc.fr

**Abstract:** With over 16,000 species recorded, ants represent a superfamily whose venoms have long remained little studied, given the small size of these insects. It has been previously demonstrated that their venom exhibits high diversity in its composition (alkaloids, proteins, peptides). Our research group has previously deciphered the venom of several ant species, and showed they were mainly composed of peptides. For now, only four of them have been physiologically characterized. Herein, we present our method used to screen the peptide bank of the laboratory over various physiological parameters. This project aims to identify new biological effects of ant venom peptides and to elucidate their underlying mechanisms of action, with a focus on non-cytotoxic peptides. First, the cytotoxic activity of peptides was assessed by using both lactate dehydrogenase-based mortality assay and formazan reduction-based viability assay. Then, the pro- or anti-proliferative effect of peptides was monitored with the same formazan reduction-based test, and the impact on migration was evaluated by using the wound-healing assay. As venom peptides frequently target membrane protein such as G protein-coupled receptors (GPCRs) and ion channels, we screened a possible effect on calcium modulation using the fluorescent dye FLUO-4/AM, cAMP production using an HTRF assay or on membrane potential using the fluorescence dye Dibac 4(3). All experiments were conducted on various human cell lines. For now, 14 peptides from the ants *Tetramorium bicarinatum*, *T*. *africanum* and *Manica rubida* were selected on their biochemical properties and tested for each of the assay developed in the lab. The cytotoxicity studies revealed that 10 peptides were non-cytotoxic. None of them showed any effects on proliferation or migration. However, we observed several peptides acting on receptor or ion channel modulation. For instance, a selective activation of a Gαq protein-coupled receptor was observed with one of the tested peptides. Indeed, it elicited a strong calcium mobilization from intracellular stocks, which was abolished in presence of the selective Gαq protein inhibitor YM-254890. This GPCR modulation was observed on non-invasive breast cancer cells (MCF-7) but was not detected on invasive ones (MDA-MB-231). The identification of the target receptor and its relevance in cancer physiopathology is currently on-going. We present in this work our method to screen our peptide library over significant physiological parameters. By applying these screening assays, we found at least a GPCR agonist among the tested peptides. These results combined to our previous studies confirm the biological interest of ant venom peptides.

**Keywords:** ant venom; ion channel; peptide; protein G

### 5.11. Assembly at Chromosome-Level of the Genome of the Ant T. bicarinatum Reveals a Tandem Organization of Venom Peptide Genes and Allows the prediction of Their Regulatory and Evolutionary Profiles

 Axel Touchard ^1^, Valentine Barasse ^2^, Jean-Michel Malgouyre ^2^, Michel Treilhou ^2^, Christophe Klopp ^3^, Elsa Bonnafé ^2,^*
 ^1^ Department of Entomology, Cornell University, 14853 Ithaca, NY, USA.
 ^2^ BTSB-EA 7417, Université de Toulouse, Institut National Universitaire Jean-François Champollion, Place de Verdun, 81000 Albi, France.
 ^3^ Unité de Mathématique et Informatique Appliquées de Toulouse, UR0875, Genotoul Bioinfo, INRAE Toulouse, 31326, Castanet-Tolosan, France.
 * Correspondence: elsa.bonnafe@univ-jfc.fr

**Abstract:** Venoms are cocktails of diverse secreted molecules used to deter predators and/or subdue prey and have evolved independently over a hundred times in the animal kingdom. Origin and diversification of venom peptide toxins is a fascinating theme, and genomic approaches are essential to better understand processes underlying their evolution. We previously showed that Myrmicinae venom is composed of several peptides that gathered into seven precursors family. Analysis of the *Tetramorium bicarinatum* genome enabling further genomic approaches was necessary to understand the processes underlying the evolution of these myrmicitoxins. Here, we report the chromosomal structure of this genome and the organisation of 44 venom peptide genes (*vpg*). The genome of *T. bicarinatum* is carried by eleven chromosomes with *vpg* organised in tandem repeats on four different chromosomes. This organisation, together with the Maximum likelihood evolutionary analysis of *vpg* sequences, is consistent with evolution by local duplication of ancestral genes for each precursor family. The structure of the *vpg* into two or three exons is conserved after duplication events while the promoter regions are the least conserved parts of the *vpg* even for genes with highly identical sequences. This suggests that enhancer sequences were not involved in duplication events, but were recruited from surrounding regions. Expression level analysis revealed that most *vpg* are highly expressed in venom glands, although one gene or group of genes is much more highly expressed in each family. Finally, the examination of the genomic data revealed that several genes encoding transcription factors are highly expressed in the venom glands. The search for binding sites of these transcription factors in the *vpg* promoters revealed hot spots of GATA sites in several *vpg* families.

**Keywords:** ant; evolution; expression; genome; peptide; toxin; venom

### 5.12. Comparison of Toxicological and Structural Recognition Data for the Detection of Tetrodotoxins in Pufferfish

 Jaume Reverté ^1^, Mounira Alkassar ^1^, Maria Rambla-Alegre ^1^, Andres Sanchez-Henao ^1^, Manolis Mandalakis ^2^, Panagiota Peristeraki ^2^, Francesc X. Sureda ^3^, Jorge Diogène ^1^, Mònica Campàs ^1,^*
 ^1^ IRTA, Ctra. Poble Nou km 5.5, 43540 La Ràpita, Spain.
 ^2^ Hellenic Centre for Marine Research, P.O. Box 2214, Heraklion, Greece.
 ^3^ Pharmacology Unit, Faculty of Medicine and Health Sciences, Universitat Rovira i Virgili, C/St. Llorenç 21, E-43201, Reus (Tarragona), Spain
 * Correspondence: monica.campas@irta.cat

**Abstract:** Tetrodotoxins (TTXs) are one of the most potent marine neurotoxins known, being responsible for many poisoning accidents and some fatalities. The pufferfish *Lagocephalus sceleratus*, the main vector of TTXs in nature, may contain different multi-TTX profiles, with the presence of a wide variety of TTX analogues. Up to now, more than 30 TTX analogues have been described but little is known about their role in poisoning. In this work, we evaluated the toxicity equivalency factors (TEFs) of five TTX analogues, purified from the liver of a pufferfish, using a single cell biosensing device based on patch clamp technology. Additionally, the cross-reactivity factors (CRFs) of these TTX analogues against an anti-TTX monoclonal antibody were evaluated using a magnetic bead-based immunoassay. Among the five TTX analogues tested, 11-deoxyTTX and 11-norTTX-6(S)-ol are the most toxic analogues and also the ones with the highest cross-reactivity. On the contrary, 5,11-dideoxyTTX, 6,11-dideoxyTTX and 5,6,11-trideoxyTTX have lower toxicities and cross-reactivities. Good correlations between both bioanalytical techniques were obtained after the application of the TEFs or CRFs to the individual TTXs contents determined by LC-MS/MS. The integration of toxicological or structural recognition data from different bioanalytical approaches allows one to better understand the results obtained from the analysis of naturally contaminated samples.

**Keywords:** automated patch clamp; electrophysiology; immunoassay; magnetic bead; TTX analogue

### 5.13. Is the Cyclic Imine Core Common to the Marine Macrocyclic Toxins Sufficient to Dictate Nicotinic Acetylcholine Receptor Antagonism?

 Yves Bourne ^1^, Gerlind Sulzenbacher ^1^, Laurent Chabaud ^2,$^, Rómulo Aráoz ^3^, Zoran Radić ^4^, Sandrine Conrod ^5,&^, Palmer Taylor ^4^, Catherine Guillou ^2^, Jordi Molgó ^3^, Pascale Marchot ^1,5,^*
 ^1^ CNRS, Aix-Marseille Univ, Lab “Architecture et Fonction des Macromolécules Biologiques” (AFMB), Faculté des Sciences Campus Luminy, 13288 Marseille, CEDEX 09, France.
 ^2^ Institut de Chimie des Substances Naturelles (ICSN), CNRS, Univ Paris-Sud, Univ Paris-Saclay, 91198 Gif-sur-Yvette France.
 ^3^ CEA, INRAE, Univ Paris-Saclay, DMTS, SIMoS, ERL CNRS, 91191 Gif-sur-Yvette, France.
 ^4^ Skaggs School of Pharmacy and Pharmaceutical Sciences (SSPPS), Univ of California San Diego, La Jolla, CA 92093-0650, USA.
 ^5^ Centre de Recherche en Neurobiologie et Neurophysiologie de Marseille (CRN2M), CNRS, Aix Marseille Univ, 13344 Marseille, France.
 ^$^ Current address: Univ Bordeaux, CNRS, Bordeaux INP, ISM, 33405 Talence, France.
 ^&^ Current address: Aix-Marseille Univ, CNRS, IRD, Collège de France, INRAE, CEREGE, 13545 Aix-en-Provence France.
 * Correspondence: pascale.marchot@univ-amu.fr

**Abstract:** Macrocyclic imine phycotoxins are an emerging class of chemical compounds associated with harmful algal blooms and shellfish toxicity. Earlier binding and electrophysiology experiments on nAChR subtypes and their soluble AChBP surrogates evidenced common trends for prominent antagonism, binding affinities, and receptor-subtype selectivity. Earlier, complementary crystal structures of AChBP complexes with phycotoxins showed that common determinants within the binding nest at each subunit interface confer high-affinity toxin binding, while distinctive determinants from the flexible loop C, and either capping the nest or extending towards peripheral subsites, dictate broad versus narrow receptor subtype selectivity [1,2,3]. From these data, small spiroimine enantiomers mimicking the functional core motif of CiTXs were chemically synthesized and characterized. Voltage-clamp analyses involving three nAChRs revealed preserved antagonism for both enantiomers, despite lower binding affinities and subtype specificity, compared with their macrocyclic relatives. Binding and structural analyses involving two AChBPs pointed to positional variability of the spiroimines along with a range of loop-C conformations, suggesting mixed agonist/antagonist properties. These data highlight the major contribution of the spiroimine core to binding within the nAChR nest and confirm the need for an extended interaction network as established by the macrocyclic toxins to define high affinities and subtype specificity [4]. Hence, this study identifies a minimal set of functional pharmacophores and binding determinants as templates for designing new effectors targeting disease-associated nAChR subtypes.

 
**References**
1. Bourne, Y.; Radić, Z.; Aráoz, R.; Talley, T.T.; Benoit, É.; Servent, D.; Taylor, P.; Molgó, J.; Marchot, P. Structural determinants in phycotoxins and AChBP conferring high affinity binding and nicotinic AChR antagonism. *Proc. Natl. Acad. Sci. USA* **2010**, *107*, 6076–6081.
2. Bourne, Y.; Sulzenbacher, G.; Radić, Z.; Aráoz, R.; Reynaud, M.; Benoit, E.; Zakarian, A.; Servent, D.; Molgó, J.; Taylor, P.; Marchot, P. Marine macrocyclic imines, pinnatoxins A and G: structural determinants and functional properties to distinguish neuronal α7 from muscle α1(2)βγδ nAChRs. *Structure* 2015, *23*, 1106–1115.
3. Molgó, J.; Marchot, P.; Aráoz, R.; Benoit, É.; Iorga, BI.; Zakarian, A.; Taylor, P.; Bourne, Y.; Servent, D. Cyclic imine toxins from dinoflagellates: a growing family of potent antagonists of the nicotinic acetylcholine receptors. *J. Neurochem*. **2017**, *142* (Suppl. 2), 41–51.
4. Bourne, Y.; Sulzenbacher, G.; Chabaud, L.; Aráoz, R.; Radić, Z.; Conrod, S.; Taylor, P.; Guillou, C.; Molgó, J.; Marchot, P. The cyclic imine core common to the marine macrocyclic toxins is sufficient to dictate nAChR antagonism. *Mar. Drugs* **2024**, in revision.

**Keywords:** acetylcholine-binding protein; binding affinity; competitive antagonism; crystal structure; cyclic imine; electrophysiology; nicotinic acetylcholine receptor; pharmacophore; receptor subtype selectivity; spiroimine

### 5.14. Conotoxins: Structural Diversity, Pharmacological Relevance, and Synthesis Strategies

 Yazid Mohamed Souf ^1,^*, Nicolas Inguimbert ^1^, Sébastien Dutertre ^2^
 ^1^ CRIOBE, UAR CNRS-EPHE-UPVD 3278, Université de Perpignan Via Domitia, 58 avenue Paul Alduy, 66860 Perpignan, France.
 ^2^ IBMM, Université Montpellier, CNRS, ENSCM, 34093 Montpellier, France.
 * Correspondence: yazid.souf@univ-perp.fr

**Abstract:** Conotoxins are disulphide-rich peptides that have been identified from cone snails’ venom. Due to their biological activity and structural diversity, they are classified into several subfamilies [1]. Each of these subfamilies can target different types of pharmacological receptors, such as nicotinic acetylcholine receptors (nAChRs), sodium channels, or G protein-coupled receptors, among others that are yet to be explored. Besides their pharmacological interest, their synthesis represents a significant challenge due to the considerable number of disulphide bridges they can contain, ranging up to five or more. These bridges provide them with a specific three-dimensional structure, thus increasing their affinity with the targeted receptor [2].


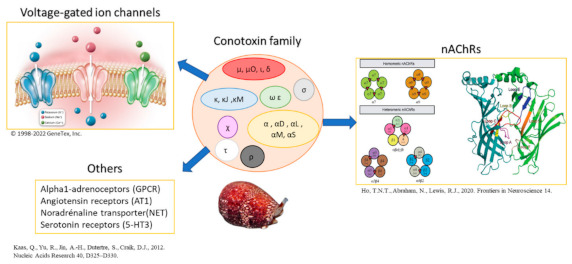


Developing versatile strategies to synthesize chemically these compounds will reveal their untapped potential. This discussion will focus on the one-pot synthesis method of these cyclic peptides and their potency and selectivity against biological receptors [3].

 
**References**
1. Jin, A.H.; Muttenthaler, M.; Dutertre, S.; Himaya, S.W.A.; Kaas Q.; Craik D.J.; Lewis R.J., Alewood P.F. Conotoxins: chemistry and biology. *Chem. Rev.* **2023**, *119*, 11510–11549.
2. Wu, Y.; Wu, X.; Yu, J.; Zhu, X.; Zhangsun, D.; Luo, S. Influence of disulfide connectivity on structure and bioactivity of α-conotoxin TxIA. *Molecules* **2014**, *19*, 966–979.
3. Laps, S.; Sun, H.; Kamnesky, G.; Brik, A. Palladium-mediated direct disulfide bond formation in proteins containing S-acetamidomethyl-cysteine under aqueous conditions. *Angew. Chem. Int. Ed.* **2019**, *58*, 5729–5733.

**Keywords:** conotoxin; pharmacological receptor; synthesis

### 5.15. Peptides for Lung Disease: Possibilities and Pitfalls

 Cliff Taggart *
 Airway Innate Immunity Research Group, Wellcome-Wolfson Institute for Experimental Medicine, School of Medicine, Dentistry and Biomedical Sciences, Queen’s University Belfast, Belfast BT1, Northern Ireland.
 * Correspondence: c.taggart@qub.ac.uk

**Abstract:** In inflammatory lung diseases, such as cystic fibrosis (CF), lung infection perpetuates a destructive cycle of inflammation resulting in progressive decline in pulmonary function. Finding new ways to target lung infection is therapeutically relevant, especially in an era of antibiotic resistance. A potential therapeutic approach is the use of antimicrobial peptides, naturally occurring peptides critical to the innate immune response. Unfortunately, therapeutic development has been hindered by poor stability, protease susceptibility, and toxicity issues. We produced synthetic peptide derivatives based on naturally occurring snake-derived peptide templates. In addition, we evaluated the therapeutic potential of nebulisable snake-derived peptides for lung disease. In initial studies, using an in vitro LPS-induced inflammation model, and antimicrobial assays, we showed that our peptides possess potent antibacterial and anti-inflammatory activities. We assessed peptide stability in CF sputum and found that the peptides were susceptible to degradation as a result of proteolysis by neutrophil elastase (NE). Further experiments using NE-incubated peptides revealed that some of these peptides retained activity. We subsequently tested the potential of a cohort of peptides for their nebulisation potential in a ‘human lung’ model. We showed that three of the peptides tested can be nebulised with retention of anti-inflammatory and anti-bacterial activities following nebulisation. To conclude, snake-derived peptides represent a potential therapeutic option for the treatment of inflammatory lung disease. Although susceptible to NE degradation, a number of these peptides retained function post-degradation and a number of peptides demonstrated potential for direct lung delivery.

**Keywords:** degradation; inflammation; lung disease; peptide

## 6. Poster Presentations (When More than One Author, the Underlined Name Is That of the Presenter)

### 6.1. β-Cyclodextrin Polymers as a Smart Agent to Capture Tetrodotoxins

 Mounira Alkassar ^1^, Jaume Reverté ^1^, Alex Fragoso ^2^, Mabel Torréns ^2^, Mirjam Klijnstr ^3^, Arjen Gerssen ^3^, Jorge Diogène ^1^, Monica Campàs ^1,^*
 ^1^ IRTA, Ctra. Poble Nou km 5.5, 43540 La Ràpita, Spain.
 ^2^ Universitat Rovira i Virgili, Avinguda Països Catalans 26, 43007 Tarragona, Spain.
 ^3^ Wageningen University & Research, P.O. Box 230, 6700 AE Wageningen, The Netherlands.
 * Correspondence: monica.campas@irta.cat

**Abstract:** Tetrodotoxins (TTXs) are a group of potent neurotoxins produced by bacteria, usually found in pufferfish but recently also detected in shellfish. These toxins may reach consumers and cause them intoxication with symptoms such as muscle weakness, respiratory failure, and ultimately death. Therefore, the detection of TTXs is of great importance in seafood safety. The cell-based assay (CBA) is commonly used to detect marine toxins because of its high sensitivity and because it provides an estimation of the composite toxicity of a sample. However, when analysing natural seafood samples, matrix effects may interfere in the CBA. In this work, five insoluble cyclodextrin polymers, with different chemicals structures (βCDPi-1.4SO3 (DS2), βCDPi-1.3SO3 (DS2), βCDPi-1.3SO3 (DS4), βCDPi-1.4SO3 (DS4) and βCDPi-NCH3 (DS5)) have been investigated as novel clean-up materials for TTXs. The best recoveries were achieved with βCDPi-1.4SO3 (DS2) (~100%). This cyclodextrin polymer had the capability of capturing as much as ~35 ng TTX/mg cyclodextrin polymer and allowed exposing cells to at least ~200 mg/mL of oyster extract. The applicability of this strategy has been demonstrated by the detection of low TTXs concentrations in shellfish extracts from The Netherlands. The use of this clean-up strategy improved the sensitivity of the assay at least 27 times. Therefore, the developed clean-up method combined with CBA is a promising strategy for the detection of low TTXs contents in shellfish, and possibly also pufferfish, which could be implemented in research and monitoring programs for seafood safety.

**Keywords:** cell-based assay; cyclodextrin polymer; shellfish; tetrodotoxin

### 6.2. Venom Analysis: from 5 Months to 5 Hours

 Luis L. Alonso ^1,^*, Jory Van Thiel ^1^, Saer Samanipour ^2^, Jeroen Kool ^1^
 ^1^ Amsterdam Institute of Molecular and Life Sciences, Division of Bioanalytical Chemistry, 1081 HV Amsterdam, The Netherlands.
 ^2^ Van’t Hoff Institute of Molecular Sciences, 1081 HV Amsterdam, The Netherlands.
 * Correspondence: l.lago.alonso@vu.nl

**Abstract:** Snakebite is considered one of the most lethal Neglected Tropical Diseases. Snake venoms are known for their complex nature and venom composition can be investigated with liquid chromatography mass spectrometry (LC-MS). The LC-MS data processing after analysis of the toxins in venoms, however, is a tedious and long process. Besides venom complexity regarding composition at the interspecies level, individual venoms at the intraspecies level also can vary heavily in terms of composition, which depends on many different factors. While venom variation between species has been well-studied, the extent of intraspecific variation is starting to gain significant interest. The main bottleneck in studying such a complex biomatrix at the chemical level (i.e., at the toxin accurate mass level) includes the manual toxin deconvolution and identification from the measured LC-MS data. This study provides a new methodology that allows us to rapidly analyse and process the measured data of numerous venom samples in an automated fashion. We verified this principle through LC-MS analysis of venoms of two genera of snakes, *Pseudonaja* and *Oxyuranus*, which include some of the most venomous snakes in the world. This was performed both manually–which took 5 months–, and automatically–which took 5 h. The automated data extraction and deconvolution of the LC-MS raw files was performed directly after analysis. Next, in-house written scripts were used to process and sort all data. Finally, the results between the automatically and the manually extracted and sorted data were compared, and we found that the same results were obtained. This workflow provides opportunities to perform fast in-depth venom variation and venom composition research. It opens new possibilities for studying animal venoms as evolutionary model systems and study venom variation in the aid of developing better antivenoms.

**Keywords:** bioanalytics; high throughput; venom variation

### 6.3. Venom Peptides: Emerging Biological Tools for Lung Cancer Diagnosis

 Romain Baudat, Pascal Kessler, Évelyne Benoit, Denis Servent *
 Université Paris-Saclay, CEA, Institut des sciences du vivant Frédéric Joliot, Département Médicaments et Technologies pour la Santé (DMTS), Service d’Ingénierie Moléculaire pour la Santé (SIMoS), EMR CNRS/CEA 9004, 91191 Gif-sur-Yvette, France.
 * Correspondence: denis.servent@cea.fr

**Abstract:** Many cancers, and the non-small cell lung cancer in particular, are associated with a disturbance of the mechanisms controlling ion homeostasis due, especially, to dysfunction and/or overexpression of many ion channels such as certain subtypes of sodium (Nav1.7) and potassium (KCa3.1) channels, pH-sensitive channels (ASICs for acid sensing ion channels) and nicotinic acetylcholine receptors (nAChRs: α7 and α9/α10 subtypes). In particular, these ion channels play an important role in cancer cell aggressiveness and tumour invasion, their overexpression likely to be correlated with the development of cancer. Although imaging approaches (radiography, CT-scan or PET-scan) are widely used in the anatomical characterization of lung cancers, some of their limitations require the development of new imaging probes, more specific of overexpressed molecular targets in lung adenocarcinomas. Peptide toxins, which have unique pharmacological properties, are well-known to bind selectively and with high affinity on certain ion channels overexpressed in cancerous cells. Therefore, a promising solution towards the development of new lung cancer biomarkers appears to be the chemical synthesis of peptide toxins allowing them to be marked by various fluorescent or radioactive probes using “click” chemistry. Preliminary results by RT-qPCR reveal the overexpression of Nav1.7, nAChRs α7 and α9 in different cancerous cell lines, results that need to be confirmed in lung cancer biopsies. Different couples of toxins/ion channels were explored notably by imaging experiments. For example, confocal microscopy reveals that α-bungarotoxin (α-BgTx), which bind to α7 and α9 nicotinic receptor subtypes are colocalized with the membrane of invasive cancer cells, while Huwentoxin-IV (HwTx-IV), which interacts with Nav1.7, is colocalized at the membrane of all lung cancerous cell lines. Additionally, electrophysiological analysis will be performed to confirm the absence of effect of toxin labelling on their functional property. Following these in vitro experiments, in vivo analysis will be performed with fluorescent or TEP imaging with xenografted mice. This study will participate to the development of new lung cancer biomarkers.

**Keywords:** diagnosis; imaging; ion channels; lung cancer; peptide synthesis

### 6.4. mCherry-Vm24: A Powerful Tool for the Visualization of the Voltage-Gated Potassium Channel Kv1.3

 Jesús Angel Borrego Terrazas *, Muhammad Umair Naseem, Amna S. Al-Olaimi, Gyorgy Panyi
 Department of Biophysics and Cell Biology, Faculty of Medicine, Research Center for Molecular Medicine, University of Debrecen, Egyetem ter. 1, 4032 Debrecen, Hungary.
 * Correspondence: jesus.borrego@med.unideb.hu

**Abstract:** The fusion of fluorophores and peptidic pore blockers generates valuable molecular tools to study the structure and function of the voltage-gated potassium channel Kv1.3, which is associated with numerous autoimmune and neurological disorders. These peptide-fluorophore chimeras improve the observation of a range of molecular processes and functions involving the Kv1.3 channel in both preclinical models and clinical research. This study presents mCherry-Vm24, a chimeric variant of the Kv1.3 pore blocking Vm24 peptide conjugated to the mCherry fluorophore. mCherry-Vm24 was produced by recombinant expression in *Pichia pastoris*. After purification through Ni-NTA affinity chromatography and size-exclusion HPLC, the resulting recombinant mCherry-Vm24 exhibits fluorescence excitation and emission profiles comparable to those previously reported for native mCherry. Electrophysiological assays demonstrate that mCherry-Vm24 binds to Kv1.3 channels with a Kd of 275 pM and maintains its selectivity over KCa3.1, Kv1.1, and Kv1.2 channels. Moreover, similar to Vm24, mCherry-Vm24 exhibits non-reversible binding properties, rendering it a fluorescent probe with almost irreversible binding to the Kv1.3 channel. In addition, using confocal microscopy, it was possible to detect the mCherry signal coming from CHO cells transfected to express Kv1.3 channels and subsequently labelled with mCherry-Vm24. These findings demonstrate the versatility of mCherry-Vm24 as a powerful tool for studying Kv1.3 channels in various protocols, such as competition assays, detection, tracking, etc.

**Keywords:** Kv1.3 labelling; pore blocker; recombinant protein

### 6.5. Ant Venom Peptides: From Screening Assays to Biological Targets Identification

 Guillaume Boy ^1^, Karthi Duraisamy ^2^, Elsa Bonnafé ^1^, Jérôme Leprince ^2^, Benjamin Lefranc ^2^, Michel Treilhou ^1^, Arnaud Billet ^1,^*
 ^1^ BTSB-EA 7417, Université de Toulouse, Institut National Universitaire Jean-François Champollion, Place de Verdun, 81012 Albi, France.
 ^2^ Inserm U1239, Différenciation et Communication Neuronale et Neuroendocrine, Normandie Univ, UNIROUEN, Plate-forme de Recherche en Imagerie Cellulaire Normandie (PRIMACEN), 76000 Rouen, France.
 * Correspondence: arnaud.billet@univ-jfc.fr

**Abstract:** Please, refer to Section 5.10.

### 6.6. Insights into the Composition of the Defensive Venom of Lycosa Tarantula

 Fabrice Saintmont, Guillaume Abbal, Claudia Bich, Sébastien Dutertre *
 IBMM, Université Montpellier, CNRS, ENSCM, 34093 Montpellier, France.
 * Correspondence: sebastien.dutertre@umontpellier.fr

**Abstract:** Spiders, like many other venomous animals, have evolved venom to rapidly incapacitate preys but also to deter aggressors. Only in the past decade was demonstrated the separate evolution of specific “predatory” and “defensive” venoms within the same animal, such as cone snails and assassin bugs. Such distinction in spiders is unknown, and therefore more work is required to investigate the composition of “injected” spider venom as opposed to dissected gland extracts or electrostimulated collected venom. Here, we report on the analysis of a venom recovered after a defensive bite and after electric stimulation of a wolf spider, *Lycosa tarantula*. LC-MS traces are remarkably similar between male and female defensive venoms, and only minor differences were noted between defensive and electrically collected venoms. *De novo* sequencing of the major peaks present in the defensive venom, in combination with our venom gland transcriptomic data, revealed seven linear peptide sequences. Remarkably, these seven peptides are processed from a single transcript, demonstrating for the first time that the defensive venom of *Lycosa tarantula* is dominated by short linear peptides rather than cysteine-rich peptides. On-going work will investigate the biological activity of these peptides.

**Keywords:** cytolytic peptide; defence; mass spectrometry; venom

### 6.7. Proteomics Reveals a Compartmentalised Venom Duct in the Two Phylogenetically Related Cone Snails Rhombiconus Fuscatus (Born, 1778) and R. Imperialis (Linnaeus, 1758)

 Antonio Caballero Foncubierta ^1,^*, Manuel Jiménez Tenorio ^2^, Rafael Zardoya ^3^, Juan Carlos Garcia Galindo ^1^
 ^1^ Universidad de Cádiz, Facultad de Ciencias, Departamento de Química Orgánica-INBIO, Puerto Real, 11006 Cádiz, Spain.
 ^2^ Universidad de Cádiz, Facultad de Ciencias, Departamento de CMIM y Química Inorgánica-INBIO, Puerto Real, 11006 Cádiz, Spain.
 ^3^ Museo Nacional de Ciencias Naturales (MNCN-CSIC), 28022 Madrid, Spain.
 * Correspondence: antonio.caballerofoncubierta@alum.uca.es

**Abstract:** *Rhombiconus fuscatus* (Born, 1778) and *R. imperialis* (Linnaeus, 1758) are two phylogenetically related cone snails present in the Indo-Pacific region that feed on polychaetes belonging to the family Amphinomidae (Lamarck, 1818) known as fireworms. Their venom duct, where conotoxins are contained in, is noticeably divided into two sections distinguished by their colour (red in the distal part from the bulb and pale yellow in the proximal region). The proteomic study of the two regions of both species was carried out using two mass spectrometric techniques: ultra-high performance liquid chromatography coupled to mass spectrometry (UHPLC-MS) and liquid chromatography coupled to tandem mass spectrometry (LC-MS/MS). Nineteen conotoxins, previously described in *R. imperialis* at a transcriptomic level, were identified using UHPLC-MS while LC-MS/MS analysis was able to identify 74 generic proteins and 65 conotoxins present in the venom duct of *R. imperialis* and/or *R. fuscatus*. The combined use of both spectrometric techniques permitted to identify 28 conotoxins from the transcriptome of *R. imperialis* in any of both species. Also, the number and intensity of the conotoxins identified by UHPLC-MS allowed us to study the differences in the venom composition according to three variables: region of the venom duct (proximal or distal), species (*R. fuscatus* or *R. imperialis*) and intraspecific variability. UHPLC-MS analysis showed a higher amount of conotoxins in the proximal part of the venom duct while the distal section was found to be mostly composed of low molecular weight non-peptide molecules suggesting an ecological significance: the proximal part contains most of the defence-evoked conotoxins while the small molecules of the distal region would be used for prey capture. Principal component analysis on the conotoxin intensity showed differences in the peptide composition of *R. fuscatus* and *R. imperialis* supporting the hypothesis that both are differentiated species although this hypothesis should be confirmed by genomic studies. It was found little intraspecific variability among the three individuals of *R. imperialis* and between the two individuals of *R. fuscatus*, which supports the hypothesis that dietary breadth is positively correlated with venom complexity in cone snails. The 28 identified conotoxins were modelled using AlphaFold2 in order to obtain their hypothetical 3D structures, which were compared against a database of other conotoxins’ tertiary structures using RUPEE. This in silico study allowed us to propose a potential molecular target for four conotoxins.

**Keywords:** cone snail; conotoxin; proteomics; *Rhombiconus fuscatus*; *Rhombiconus imperialis*

### 6.8. Improvements in Methodology for Affinity Purification of Peptide Toxins from Crude Venoms Using Various Cell Membrane Receptors

 Lou Freuville ^1^, Rudy Fourmy ^2^, Aude Violette ^2^, Rómulo Aráoz ^3^, Nicolas Gilles ^3^, Alain Brans ^4^, Loïc Quinton ^1,^*
 ^1^ Mass Spectrometry Laboratory, MolSys Research Unit, Department of Chemistry, University of Liège, Allée du Six Août, 11–Quartier Agora, 4031 Liège, Belgium.
 ^2^ Alphabiotoxine Laboratory, 7911 Montroeul-au-Bois, Belgium.
 ^3^ Université Paris-Saclay, CEA, Département Médicaments et Technologies pour la Santé (DMTS), Service d’Ingénierie Moléculaire pour la Santé (SIMoS), Equipe Mixte de Recherche CNRS/CEA (EMR 9004), 91191 Gif sur Yvette, France.
 ^4^ Centre for Protein Engineering, University of Liège, B-4000 Liège, Belgium.
 * Correspondence: loic.quinton@uliege.be

**Abstract:** Animal venoms are complex chemical cocktails containing a wide range of biologically active peptides whose selectivity and efficiency act against membrane targets, such as G-protein coupled receptors (GPCRs). Such toxins are exploited for the deep study of their associated receptors and in pharmacophore modelling to identify leads for the development of the drugs of tomorrow. The scope of venoms for drug discovery is rapidly emerging but is mostly undone, mainly due to the complexity of venoms and the low throughput to screen venoms towards multiple molecular receptors of interest. In this context, our work proposes an innovative methodology of toxin selection based on affinity capture on cellular membranes monitored by mass spectrometry. The first selected target is the type 2 vasopressin receptor (hV2R), a GPCR associated with vasopressin, its endogenous ligand, and with mambaquaretin-1, a toxin isolated from the venom of the green mamba, *Dendroaspis angusticeps*. The second selected target is the small conductance calcium-activated channel (SK2 and SK3 subtypes), whose well-known ligand is the toxin apamin, from *Apis mellifera* venom. Finally, our work focuses on nicotinic acetylcholine receptors (nAChRs). Cone snail venoms are rich in toxins targeting nAChRs, as it is evaluated that at least one conotoxin virtually targets nAChRs in every cone snail venom studied [1]. Membranes overexpressing the receptor of interest were incubated with either crude or pre-purified venom for screening. Toxins displaying affinities for the receptor bind to the membranes, while those without affinity are retained in solution. The mixture containing the candidates is analysed by MALDI mass spectrometry to identify the potential ligands directly from the mixtures. Positive controls were ensured using a control ligand, whereas cell membranes that do not overexpress our receptor of interest were used for negative controls. The results of this study demonstrate the effectiveness of the methodology of fishing peptides across various types of receptors. Although it is working, achieving a high-throughput strategy is the ultimate goal. Automation using magnetic beads coated with receptors to automatically capture ligands from complex mixtures will soon be considered. These improvements will make it possible to study a large number of potential peptides present in venoms and many different receptors.

 
**Reference**
1. Lebbe, E.K.; Peigneur, S.; Wijesekara, I.; Tytgat, J. Conotoxins targeting nicotinic acetylcholine receptors: an overview. *Mar. Drugs* **2014**, *12*, 2970–3004.

**Keywords:** affinity capture; animal venom; MALDI mass spectrometry

### 6.9. Trimming the Disordered C-Terminal Extension of the Protease Inhibitor Hg1 Results in a Potent Kv1.3 Blocker

 Chandamita Saikia ^1^, Hagit Altman-Gueta ^2^, Shachar Fine ^1^, Orly Dym ^3^, Felix Frolow ^2^ Eitan Reuveny ^1^, Izhar Karbat ^1,^*
 ^1^ Department of Biomolecular Sciences, Weizmann Institute of Science, Rehovot 76100, Israel.
 ^2^ Department of Plant Molecular Biology and Ecology, Tel-Aviv University, Tel-Aviv 69978, Israel.
 ^3^ Structural Proteomic Unit, Weizmann Institute of Science, Rehovot 76100, Israel.
 * Correspondence: izhar.karbat@weizmann.ac.il

**Abstract:** Animal venom contains a mixture of toxic polypeptides that act in synergy to enable the fast immobilization of prey or deter predators and competitors. This polypeptide arsenal is being constantly replenished by the recruitment of protein scaffolds from various organs into the venom gland. The recruited proteins often retain their ancestral functions, but acquire in addition specific modifications associated with novel toxic functions. Here we report an in-depth analysis of Hg1, a Kunitz-fold protease inhibitor with moderate blocking activity on Kv1.3, isolated from the venom gland of the *Hadrurus gertschi* scorpion. Electrophysiological analyses, supported by MD simulations, suggested that in contrast to canonical K^+^ blocking peptides, Hg1 does not insert a lysine side chain into the channel pore. The crystal structure of the toxin revealed an unusually long, disordered extension at the C-terminal region of the molecule. Deletion of the C-terminal extension increased the potency of the toxin by order of magnitude and restored the voltage sensitivity of the block, a hallmark of the canonical blocking mechanism. In silico analysis supported the notion that removal of the disordered C-terminal extension has lifted a geometric restraint that prevents the wild-type toxin from effectively blocking Kv1.3. We propose that Hg1 is an evolutionary intermediate between a highly potent protease inhibitor and a highly potent K^+^ channel blocker.

**Keywords:** electrophysiology; K^+^ blocker; Kunitz fold; molecular dynamics

### 6.10. High Throughput Snake Venom Glycoproteomics

 Amalia Kontochristou *, Jeroen Kool, Melissa Bärenfänger, Julien Slagboom
 Amsterdam Institute of Molecular and Life Sciences, Division of BioAnalytical Chemistry, Vrije Universiteit Amsterdam, 1081 HV Amsterdam, The Netherlands.
 * Correspondence: a.kontochristou@vu.nl

**Abstract:** Snakebite envenoming is considered a neglected tropical disease responsible for more than 100,000 deaths annually. Snake venoms are significantly complex and diverse proteinaceous mixtures that provoke a wide range of pathologies, including haemotoxicity. Snake venom toxins, mainly responsible for the activity of snake venoms, contain a great number of post-translational modifications (PTMs), among them N-glycosylation. N-glycosylation of proteins is known to be related to protein folding and function; however, there is little understanding of how N-glycosylation impacts the venom proteome. This study aimed to develop a high-throughput venom glycoproteomics methodology for screening snake venom toxins for N-glycosylation, characterising the effect of N-glycosylation on snake venom haemotoxicity and analysing N-glycans released from glycosylated snake venom toxins. For this purpose, the venoms of three snakes from the Viperidae family (*Cerastes cerastes*, *Crotalus atrox* and *Echis ocellatus*) were first separated using reverse-phase liquid chromatography and analysed with mass spectrometry in parallel to nano-fractionation onto 384 well plates. The snake venom toxins were identified using bottom-up proteomics, and the profiling of the coagulopathic activity of snake venoms was achieved using a plate-reader based plasma coagulation bioassay. Our findings confirmed the presence and diversity of N-glycans on snake venom toxins and revealed that N-glycosylation can affect the heamotoxic activity of snake venom toxins. The analytical approach developed in this study could be a valuable tool for screening snake venom toxins for N-glycosylation, enabling a better understanding of the effect of glycosylation on venom haemotoxicity.

**Keywords:** coagulation assay; mass spectrometry; N-glycosylation; toxin; viper venom

### 6.11. Tools for Sampling and Screening Ostreopsis and Its Associated Toxins in the Benthic Compartment

 Lucile Le Fresne ^1,^*, Gilbers Romero Suarez ^1^, Kylene Flynn ^1^, Guillaume Barnouin ^1^, Zoé Arrigoni ^1,2^, Rodolphe Lemee ^3^, Luisa Mangialajo ^1^, Marie-Yasmine Bottein ^1^
 ^1^ Université Côte d’Azur, CNRS, ECOSEAS, 06108 Nice, France.
 ^2^ Université de Montpellier, UFR Pharmacie, 163 rue Auguste Broussonnet, 34090 Montpellier, France.
 ^3^ Laboratoire d’Océanographie de Villefranche (LOV) UMR 7093, Sorbonne Université–CNRS 181 chemin du Lazaret, 06230 Villefranche-sur-mer, France.
 * Correspondence: lucile.le-fresne@univ-cotedazur.fr

**Abstract:** Globally and in the Mediterranean Sea, recurrence of toxin-producing benthic *Ostreopsis* cf. *ovata* blooms presents a pressing “One health” concern, by threatening human, animal and environmental health. The project aims to enhance our understanding of the distribution of the palytoxin (PLTX)-group toxins produced by *Ostreopsis* cf. *ovata* within benthic communities and habitats, and thus better comprehend the transfer of toxins up the food web. This poster introduces sampling techniques, such as light traps and artificial substrate, designed to effectively collect benthic organisms from various substrates such as sediments, canopy-forming macroalgae, seaweeds and turf macroalgae. The primary objective being to facilitate the enumeration of *Ostreopsis* cf. *ovata* cells (in both their vegetative and cyst forms) and the measurement of PLTX-group toxins concentrations. The sampling takes place in Villefranche-sur-mer (France), a strategically chosen station due to the availability of a long-term data series of *Ostreopsis* cf. *ovata* blooms. We prioritized the application of non-invasive and environmentally friendly sampling techniques, ensuring to retrieve devices following each sampling event.

**Keywords:** harmful algal bloom; one health; trophic transfer; toxin

### 6.12. Engineering and Design of Snake Venom Toxins and Binders for Novel Snakebite Therapy

 Srikanth Lingappa ^1,2^, Andrea Gonzalez-Munoz ^2^, Hannah Bolt ^2^, Christiane Berger-Schaffitzel ^1^, Fabio Parmeggiani ^1^, Imre Berger ^1,^*
 ^1^ School of Biochemistry, University of Bristol, Bristol BS8 1TD, United Kingdom.
 ^2^ AstraZeneca, Cambridge CB2 0AA, United Kingdom.
 * Correspondence: imre.berger@bristol.ac.uk

**Abstract:** Snakebites are a significant global health issue, causing over 100,000 deaths and 400,000 cases of disability annually. The World Health Organization has classified snakebite as the neglected tropical disease. Although antivenom therapies exist, their effectiveness is limited due to various factors. One major challenge is the scarcity of snake venom required for conducting experiments aimed at improving the quality of antivenom. On the other hand, recombinant production of some venom proteins can be challenging because of their complex folding and numerous disulphide bonds. Additionally, these venom proteins can be toxic to the cells expressing them. To address these challenges, we implemented a protein design strategy leveraging cutting-edge deep learning programs like AlphaFold, ProteinMPNN, and PyRosetta. This approach aims to engineer complex venom proteins, improving their expression and solubility while preserving their structural conformation. Our successful engineering and expression of active snake venom metalloproteinase in *Escherichia coli* demonstrate the efficacy of this strategy. Currently, we are conducting experiments to characterize the engineered SVMP and employing high-throughput screening to identify antibody binders for potential use in antivenom therapy.

**Keywords:** AlphaFold2; snakebite therapy; snake venom metalloproteinase

### 6.13. Is the Cyclic Imine Core Common to the Marine Macrocyclic Toxins Sufficient to Dictate Nicotinic Acetylcholine Receptor Antagonism?

 Yves Bourne ^1^, Gerlind Sulzenbacher ^1^, Laurent Chabaud ^2,$^, Rómulo Aráoz ^3^, Zoran Radić ^4^, Sandrine Conrod ^5,&^, Palmer Taylor ^4^, Catherine Guillou ^2^, Jordi Molgó ^3^, Pascale Marchot ^1,5,^*

**Author affiliations:** Please refer to Section 5.13.

**Abstract:** Please refer to Section 5.13.

### 6.14. Identification of a Mitochondrial Variant of the TRPV1 Channel Using Resiniferatoxin: Involvement in the Regulation of Ca^2+^ and Mitochondrial Temperature

 Florian Beignon ^1^, Sylvie Ducreux ^2^, Dominique Chrétien ^3^, Paule Bénit ^3^, Pierre Rustin ^3^, Malgorzata Rak ^3^, Hélène Tricoire-Leignel ^1^, Guy Lenaers ^1^, César Mattei ^1,^*
 ^1^ University of Angers, MITOVASC, Equipes CarMe and Mitolab, SFR ICAT, INSERM, CNRS, 49000 Angers, France.
 ^2^ CarMeN Laboratory, INSERM, INRA, INSA Lyon, Claude Bernard University Lyon 1, 69029 Bron, France.
 ^3^ Université Paris Cité, INSERM U1141, NeuroDiderot, 75019 Paris, France.
 * Correspondence: cesar.mattei@univ-angers.fr

**Abstract:** Mitochondria play multiple functions referred as the hub of cellular metabolism. Among them, they are at the crossroad between thermogenesis, ATP production and Ca^2+^ homeostasis. Recent data show that their inner temperature could reach ~50 °C. We hypothesized that they have a temperature control system in the form of a molecular thermostat. We identified a human variant of the Transient Receptor Potential Vanilloid 1 (TRPV1), encoding for a 150 amino-acid mitochondrial targeting sequence (MTS) at its N-terminus, followed by the six transmembrane domains and the C-terminus of TRPV1. This human mitoTRPV1 is specifically expressed in the mitochondria inner membrane and is thought to be present in mammal genomes. We took advantage of the highly specific TRPV1 agonist resiniferatoxin (RTX) to evidence the functions associated with mitoTRPV1. First, the activation of mitoTRPV1 induced a Ca^2+^ increase in the cytoplasm of transfected HEK293 cells. More specifically, RTX enhanced a Ca^2+^ efflux from the mitochondria in hot spots. This effect on Ca^2+^ homeostasis was observed in parallel with a significant reduction in the mitochondrial temperature, assessed with a mitochondrial temperature-sensitive dye. We also observed that this limitation of mitochondria overheating does not modify the respiratory parameters or the production of ATP. Interestingly, the expression of a mitoTRPV1 mutant (G684V) in HEK293 cells impaired the Ca^2+^ efflux and the temperature cooling. This rare G684V variant has been associated to exertional heat stroke, a severe disease in which patients cannot control their inner temperature in constraining conditions (hot temperature, humidity, etc.). Our data identified a new mitochondrial channel involved in the control of Ca^2+^ and temperature and opened new avenues in the understanding of the thermogenesis/thermolysis balance. Future developments could imply the use of TRPV1-targeting toxins directed to the mitochondria to pharmacologically regulate the body temperature.

**Keywords:** Ca^2+^ homeostasis; mitochondria; resiniferatoxin; temperature; TRPV1

### 6.15. 3,4-Diaminopyridine Antagonizes the Block of Muscle Nicotinic Acetylcholine Receptor Caused by Cyclic Imine Toxins at Isolated Mouse Diaphragm Preparations

 Nicolas Lamassiaude ^1^, Évelyne Benoit ^1^, Rómulo Aráoz ^1^, Armen Zakarian ^2^, Denis Servent ^1^, Jordi Molgó ^1,^*
 ^1^ Université Paris-Saclay, CEA, Département Médicaments et Technologies pour la Santé (DMTS), Service d’Ingénierie Moléculaire pour la Santé (SIMoS), Equipe Mixte de Recherche CNRS/CEA (EMR 9004), 91191 Gif-sur-Yvette, France.
 ^2^ University of California Santa Barbara, Department of Chemistry and Biochemistry, Santa Barbara, CA 93106, USA.
 * Correspondence: jordi.molgo@cea.fr

**Abstract:** Cyclic imine toxins (CiTXs) are a family of worldwide-distributed marine macrocyclic phycotoxins comprising different groups of lipophilic compounds: gymnodimines (GYMs), spirolides, pinnatoxins (PnTXs), pteriatoxins (PteTXs), portimines, prorocentrolides and spiroprorocentrimine. CiTXs and the acyl derivative products of shellfish metabolism contaminate and bioaccumulate in filter-feeding bivalves, edible mollusks and other marine invertebrates and thus, represent a risk for shellfish consumers and a menace for wildlife conservation. All CiTXs studied interact and block, with different affinities, embryonic (α12βγδ) and mature (α12βεδ) nicotinic acetylcholine receptors (nAChRs) expressed in skeletal muscles. 3,4-Diaminopyridine (3,4-DAP) is a well-known K+-channel blocker that prolongs the presynaptic action potential and indirectly enhances the entry of Ca2+ into nerve terminals and, thereby, increases quantal ACh release at the neuromuscular junction. 3,4-DAP is used effectively to treat muscular weakness in various human myasthenic syndromes. The aim of this work was to determine whether 3,4-DAP, which increases quantal ACh release, is able to reverse the action of CiTXs on skeletal muscle expressing α12βεδ nAChRs. The use of isolated mouse phrenic-nerve-hemi-diaphragm muscle preparations enabled monitoring nerve-evoked isometric muscle contraction in the presence of GYM-A, PnTX-A, PnTX-G and PteTX-1 and, afterwards, the action of 3,4-DAP (10–100 µM) in the continuous presence of the respective CiTXs. The results show that 3,4-DAP improves neuromuscular function in a time- and concentration-dependent manner and, at the highest concentration used (100 µM), completely reverses the neuromuscular block caused by the CiTX studied. These results support the view that GYM-A, PnTX-A, PnTX-G and PteTX-1 are competitive and reversible inhibitors of muscle-type α12βεδ nAChRs. In fact, CiTXs can be typified as competing with the binding of ACh (the natural ligand) to the nAChR. The ability of 3,4-DAP to antagonize the action of CiTXs is related to the increased release of ACh that is able to displace the respective CiTX from their nAChR binding sites.

**Keywords:** cyclic imine toxin; 3,4-diaminopyridine; K^+^-channel block; muscle contraction; neuromuscular junction; nicotinic acetylcholine receptor; nerve terminal

### 6.16. The Molecular Mechanism of Snake Short-Chain α-Neurotoxin Binding to Muscle-Type Nicotinic Acetylcholine Receptors

 Mieke Nys ^1,^*, Eleftherios Zarkadas ^2,3^, Marijke Brams ^1^, Aujan Mehregan ^1^, Kumiko Kambara ^4^, Jeroen Kool ^5^, Nicholas R. Casewell ^6^, Daniel Bertrand ^4^, John E. Baenziger ^7^, Hugues Nury ^2^, Chris Ulens ^1^
 ^1^ Laboratory of Structural Neurobiology, Department of Cellular and Molecular Medicine, Faculty of Medicine, KU Leuven, 3000 Leuven, Belgium.
 ^2^ Université Grenoble Alpes, CNRS, CEA, IBS, 38000 Grenoble, France.
 ^3^ Université Grenoble Alpes, CNRS, CEA, EMBL, ISBG, 38000 Grenoble, France.
 ^4^ HiQscreen, Route de Compois 6, 1222 Vésenaz, Geneva, Switzerland.
 ^5^ Amsterdam Institute of Molecular and Life Sciences, Division of BioMolecular Analysis, Vrije Universiteit Amsterdam, 1081 HV Amsterdam, The Netherlands.
 ^6^ Centre for Snakebite Research & Interventions, Liverpool School of Tropical Medicine, L3 5QA Liverpool, United Kingdom
 ^7^ Department of Biochemistry, Microbiology and Immunology, University of Ottawa, Ottawa, ON, K1H 8M5, Canada.
 * Correspondence: mieke.nys@kuleuven.be

**Abstract:** Bites by elapid snakes (e.g., cobras) can result in life-threatening paralysis caused by venom neurotoxins blocking neuromuscular nicotinic acetylcholine receptors. Here, we determine the cryo-EM structure of the muscle-type *Torpedo* receptor in complex with ScNtx, a recombinant short-chain α-neurotoxin. ScNtx is pinched between loop C on the principal subunit and a unique hairpin in loop F on the complementary subunit, thereby blocking access to the neurotransmitter binding site. ScNtx adopts a binding mode that is tilted toward the complementary subunit, forming a wider network of interactions than those seen in the long-chain α-bungarotoxin complex. Certain mutations in ScNtx at the toxin-receptor interface eliminate inhibition of neuronal α7 nAChRs, but not human muscle-type receptors. These observations explain why ScNtx binds more tightly to muscle-type receptors than neuronal receptors. Together, these data offer a framework for understanding subtype-specific actions of short-chain α-neurotoxins and inspire strategies for design of new snake antivenoms.

**Keywords:** alpha-neurotoxin; nicotinic acetylcholine receptor; structure-function

### 6.17. Characterization of the Venom of Conus Canonicus from Mayotte Island

 Zahrmina Ratibou ^1,^*, Sébastien Dutertre ^2^, Nicolas Inguimbert ^1^
 ^1^ CRIOBE, UAR CNRS-EPHE-UPVD 3278, Université de Perpignan Via Domitia, 58 avenue Paul Alduy, 66860 Perpignan, France.
 ^2^ IBMM, Université Montpellier, CNRS, ENSCM, 34093 Montpellier, France.
 * Correspondence: zahrmina.ratibou@univ-perp.fr

**Abstract:** Cone snails are carnivorous marine predators that prey on molluscs, worms, or fish. They are constituted with a venom gland that produces a highly diversified venom, mainly composed of small cysteine-rich peptides called conotoxins. They produce and purposefully inject different types of venom, whether they prey or defend themselves against predators. In this study, we focus on the venom of one molluscivorous cone, known as *Conus canonicus*, collected in the Mayotte island in the Indian Ocean. Transcriptomics studies enabled identification of 99 conotoxin sequences from 21 gene superfamilies, with the most expressed sequences belonging to the O, M, conkunitzin and T superfamilies. The composition of the predatory venom will be further investigated and compared with the distal, central, and proximal sections of the venom duct to reveal its origins. These results further expand our understanding of venom-ecology relationships in cone snails and provide a novel resource for potential drug discovery programs. This study takes part in my thesis work, which aims at characterizing and comparing cone snail venoms from Mayotte and French Polynesia.

**Keywords:** conotoxin; *Conus canonicus*; transcriptomics

### 6.18. Venomics and Antivenomics from the European Addovenom Project, Towards a New Generation of Antivenoms Based on Virus Like-Particules

 Damien Redureau ^1^, Fernanda Gobbi Amorim ^1^, Thomas Crasset ^1^, Dominique Baiwir ^2^, Stefanie Menzies ^3^, Nicholas R. Casewell ^3^, Loïc Quinton ^1,^*
 ^1^ Laboratory of Mass Spectrometry, MolSys Research Unit, University of Liège, 4000 Liège, Belgium. AstraZeneca, Cambridge CB2 0AA, United Kingdom.
 ^2^ GIGA Proteomics Facility, University of Liège, 4000 Liège, Belgium.
 ^3^ Centre for Snakebite Research and Interventions, Liverpool School of Tropical Medicine, Pembroke Place, Liverpool L3 5QA, United Kingdom.
 * Correspondence: loic.quinton@uliege.be

**Abstract:** Snakebite is a neglected tropical disease causing more than 120,000 deaths a year and 3–4 times as many disabilities. Antivenom, obtained from the serum of hyperimmunised animals, remains the standard treatment. Nevertheless, antivenoms are not always effective, have undesirable side effects and are expensive to produce. ADDovenom, a multidisciplinary European project, aims to bring about a rapid change in the treatment of snakebites using ADDomer©, a thermostable synthetic virus-like particle with high affinity, designed to rapidly eliminate deleterious toxins from the bloodstream after envenomation. The ADDovenom project is exploiting the potential of advanced mass spectrometry and proteomics to inventory the toxin repertoire of nine of the most deadly snakes in sub-Saharan Africa, in order to analyse the effectiveness of the ADDomer technology. Five species of *Dendroapis* and four species of *Echis* were selected. Proteomics of the venoms (10 ug of each) was carried out using an innovative methodology named Multi-Enzymatic Limited Digestion—MELD. Venoms were analysed using an Acquity M-Class coupled to a Q-Exactive™ mass spectrometer (Top 12). Protein identification and relative quantification were performed by Peaks Studio X+, using the venom gland transcriptomes. The nine venoms contain compounds with a wide range of molecular weights, mainly from 1 to 120 kDa. *Dendroaspis* species had a majority of large peptides in the 6–15 kDa range, while *Echis* species were found to be rich in higher molecular weight proteins (up to 120 kDa). Proteomics results showed that around 46% of *Dendroaspis* venoms were composed of toxins, with 3-finger toxins, Kunitz-type toxins and snake venom metalloproteinase being the most highly expressed toxin classes. *Echis* contained almost 49% of toxins and proteases, with snake venom metalloproteinases, C-type lectin-like proteins and serine proteinases being the most expressed. A total of 62 peptides were found in common among *Dendroaspis* species and 103 peptides were shared in all *Echis* species. These antigens can be subjected to bioinformatics modelling (AlphaFold) to identify their position in the 3D structure of complete toxins. In this study, a preliminary assessment of the potency of antivenoms is also proposed. This method involves the immunocapture of venom toxins by antivenom antibodies grafted onto magnetic beads. This approach will not only provide a better understanding of the mode of action and efficacy of various antivenoms, but will also enable them to be compared with our innovative ADDobodies/ADDomers constructs.

**Keywords:** ADDovenom; antivenom; mass spectrometry; venom

### 6.19. Proteomic Insights into Apis Mellifera Syriaca Venom and Its Influences on Murine Splenic Cytokines Profile

 Christina Sahyoun ^1,2^, Miriam Khoury ^3^, Jacinthe Frangieh ^1,2^, César Mattei ^1^, Ziad Fajloun ^2,4^, Mark Karam ^3^, Christian Legros ^1,^*
 ^1^ Univ Angers, MITOVASC, Equipe CarMe, SFR ICAT, INSERM, CNRS, 49000 Angers, France.
 ^2^ Lebanese University, Laboratory of Applied Biotechnology (LBA3B), Azm Center for Research in Biotechnology and Its Applications, 1300 Tripoli, Lebanon.
 ^3^ University of Balamand, Faculty of sciences, Al-koura campus, Lebanon.
 Lebanese University, Department of Biology, Faculty of Sciences 3, 1300 Tripoli, Lebanon.
 * Correspondence: christian.legros@univ-angers.fr

**Abstract:** Honeybee venoms are renowned for their noteworthy biological activities. Upon a bee sting from the *Apis mellifera* species, an immediate local inflammatory reaction is manifested while in allergic patient a bee sting could lead to more severe complications such as life-threatening anaphylactic shock. On the other hand, bee stings and bee venom injections have been used for a long time in traditional medicine to treat chronic inflammatory conditions such as rheumatoid arthritis. These opposite effects of bee venoms support the idea that they contain both pro- and anti-inflammatory molecules capable of modulating the immune response. *A. m. syriaca* is the honeybee subspecies endemic to the Middle Eastern region including Lebanon, Syria, Jordan, and Iraq. Notably, the venom of *A. m. syriaca* is poorly studied and its proteomic composition is not yet known. Nevertheless, recent research revealed some of its intriguing biological activities, including anticoagulant effects, cytotoxic effects on cancer cell lines, and anti-microbial effects. In this work, we investigated the immunomodulatory effects of the venom by evaluating the splenic levels of both pro- and anti-inflammatory cytokines in mice spleens by ELISA. First, we revealed that the venom exhibits mild toxicity in BALB/c mice following intraperitoneal injection with an LD50 of 3.8 mg/kg. Interestingly, we revealed that the *A. m. syriaca* venom at 1 mg/kg induced a decrease in IFN-γ, TNF-α, IL-4, and IL-10 at 24h post-injection. At a higher dose (3 mg/kg), an increase in IFN-γ and IL-4 levels was observed, while the levels of TNF-α and IL-10 remained low compared with the control. To better understand the immunomodulatory effects of *A. m. syriaca* venom, we conducted a comprehensive analysis of its composition using LC-ESI-MS to identify and characterize its components. Like many bee venoms from the *A. mellifera* species, the most abundant components of *A. m. syriaca* venom were shown to be mellitin and phospholipase A2, both of which have been associated with pro- and anti-inflammatory responses induced by bee venoms. At lower abundances, we detected the presence of peptides such as apamin, MCD-peptide and hyaluronidase. Altogether, these preliminary data suggest that *A. m. syriaca* venom exhibits both pro- and anti-inflammatory effects depending on the administered doses while the exact molecules behind these responses and their mode of action remain to be identified.

**Keywords:** *Apis mellifera syriaca*; cytokine; immunomodulation; melittin; phospholipase A2.

### 6.20. Towards Combinations of Human Monoclonal Antibodies from Venom-Immunized Transgenic Mice

 Julien Slagboom ^1^, Mieke Veldman ^2^, Abigail H. Lewis ^1^, Wietse Schouten ^1^, Rien Van Haperen ^2^, Freek J. Vonk ^1^, Nicholas R. Casewell ^3^, Frank Grosveld ^2^, Dubravka Drabek ^2^, Jeroen Kool ^1,^*
 ^1^ Amsterdam Institute of Molecular and Life Sciences, Division of BioAnalytical Chemistry, Vrije Universiteit Amsterdam, 1081 HV Amsterdam, The Netherlands.
 ^2^ Department of Cell Biology and Genetics, Faculty of Medicine, Erasmus Medical Center Rotterdam, 1081 HV Amsterdam, The Netherlands.
 ^3^ Centre for Snakebite Research and Interventions, LSTM, Liverpool L3 5QA, United Kingdom.
 * Correspondence: j.kool@vu.nl

**Abstract:** Human monoclonal antibodies directed against venom toxins are emerging as candidates for new snakebite treatments and diagnostics. Because of venom variability within and between species, formulations of combinations of human antibodies are proposed. We used both Harbour H2L2 and HCAb transgenic mouse platforms for different immunization campaigns. Classical monoclonal antibodies were obtained by hybridoma technology from the H2L2 platform and fully human “heavy chain only” antibodies (HCAbs) were obtained by screening HEK 293 cells libraries expressing heavy chain variable region repertoires from HCAb immunized mice. Crude venom-ELISAs were employed for initial toxin binding antibody selection. To identify which toxin bound to each monoclonal antibody, we developed and applied an analytical screening method employing antibody-toxin fishing from crude venoms in 384 filter-well plate format. The approach uses Protein G beads for antibody capture followed by exposure to crude venom allowing specific toxins to bind. This is followed by a washing/centrifugation step to remove non-binding toxins and is then followed by in-well tryptic digestion. Resulting peptides from each well are then analysed by rapid nanoLC-MS/MS peptide mass fingerprinting to identify the bound toxin(s) for each antibody under investigation. Direct sampling from high-density well plate-based hybridoma cultures worked efficiently and allowed for over 200 hybridomas to be tested per day with their antibody-toxin binder(s) as resulting outcome. R-scripts enabled automated data sorting thereby yielding Excel-lists of all hybridomas tested with their toxin binder(s). Combinations of antibodies, each binding another venom toxin, are currently being selected for further investigation towards their therapeutic value. The VH domains of HCAbs are particularly interesting from the engineering point of view for creating multi-specific agents that can target more than one toxin simultaneously.

**Keywords:** antivenom; human antibody; immunized transgenic mice; venomics

### 6.21. Synthesis and Biological Activity of Novel α-Conotoxins Derived from Endemic Polynesian Cone Snails

 Yazid Mohamed Souf ^1,^*, Gonxhe Lokaj ^2^, Veeresh Kuruva ^3^, Yakop Saed ^3^, Delphine Raviglione ^1^, Ashraf Brik ^3^, Annette Nicke ^2^, Nicolas Inguimbert ^1^, Sébastien Dutertre ^4^
 ^1^ CRIOBE, UAR CNRS-EPHE-UPVD 3278, Université de Perpignan Via Domitia, 66100 Perpignan, France.
 ^2^ Faculty of Medicine, Walther Straub Institute of Pharmacology and Toxicology, Ludwig Maximilian, University of Munich, Nußbaumstraße 26, 80336 Munich, Germany.
 ^3^ Schulich Faculty of Chemistry, Technion-Israel Institute of Technology, Israel.
 ^4^ IBMM, Université Montpellier, CNRS, ENSCM, 34093 Montpellier, France.
 * Correspondence: yazid.souf@univ-perp.fr

**Abstract:** α-Conotoxins are well-known probes for the characterization of the various subtypes of nicotinic acetylcholine receptors (nAChRs). Identifying new α-conotoxins with different pharmacological profiles can provide further insights into the physiological or pathological roles of the numerous nAChR isoforms found at the neuromuscular junction, the central and peripheral nervous systems, and other cells such as immune cells. This study focuses on the synthesis and characterization of two novel α-conotoxins obtained from two species endemic to the Marquesas Islands, namely *Conus gauguini* and *C. adamsonii*. Both species prey on fish, and their venom is considered a rich source of bioactive peptides that can target a wide range of pharmacological receptors in vertebrates. Here, we demonstrate the versatile use of a one-pot disulphide bond synthesis to achieve the α-conotoxin fold [Cys 1-3; 2-4] for GaIA and AdIA, using the 2-nitrobenzyl (NBzl) protecting group of cysteines for effective regioselective oxidation. The potency and selectivity of GaIA and AdIA against rat nicotinic acetylcholine receptors were investigated electrophysiologically and revealed potent inhibitory activities. Overall, this study contributes to a better understanding of the structure–activity relationships of α-conotoxins, which may help in the design of more selective tools.

**Keywords:** conotoxin; nicotinic acetylcholine receptor; peptide synthesis; two-electrode voltage clamp

### 6.22. A Snake Toxin Engineered into the first AVPR2-Selective PET Radiotracer Allows Specific Imaging of Ectopically Expressed AVPR2 Receptor In Vivo

 Goran Stanajic Petrovic ^1,2,^*, Pascal Kessler ^1^, Dimitri Kerselidze ^2^, Christiane Mendre ^3^, Charles Truillet ^2^, Nicolas Gilles ^1^
 ^1^ Service d’Ingénierie Moléculaire pour la Santé (SIMoS), CEA de Saclay, Université Paris-Saclay, 91191 Gif-sur-Yvette, France.
 ^2^ BioMaps, Service Hospitalier Frédéric Joliot, CEA, 91400 Orsay, France.
 ^3^ Institut de Génomique Fonctionnelle, Université de Montpellier, UMR 5203 CNRS, U1191 INSERM, 34000 Montpellier, France.
 * Correspondence: goran.stanajicpetrovic@cea.fr

**Abstract:** Many obstacles lay in the way of a disease-free world, one of them being cancers. Who does not know someone fighting against it? One’s odds would be greatly improved by more sensitive detection methods. Such a technique is here investigated which would revolutionize non-renal, type 2 vasopressin receptor (AVPR2)-expressing tumors by using PET imaging along with the first AVPR2-selective radiotracer. PET provides high sensitivity, whole body functional images and allows both radiotracer evaluation and AVPR2 investigation in vivo. Certain cancerous cells, including ccRCC metastatic ones -yet undetectable at early stages- seem to express AVPR2. This biomarker being only expressed by collecting ducts in healthy subjects represents an interesting target to detect early, kidney-distant tumours. A high-affinity, high-selectivity AVPR2 ligand was discovered several years ago: a peptide found in *Dendroaspis angusticeps* venom and called mambaquaretin (MQ). It has an ability to be transformed into a highly selective, PET-compatible radiotracer by labeling it with either 89Zr or 18F while retaining a nanomolar affinity for both human and murine AVPR2. Natural occurring MQ, MQWT, and an engineered version, MQ232, were evaluated in rodent models. The in vivo pharmacokinetic behaviour of 89Zr-labeled peptides was evaluated using PET imaging and *ex-vivo* biodistribution techniques, their plasmatic half-lives being 90 and 30 min, respectively. Both demonstrated a high selectivity for the kidneys with low non-specific signal. Tumour labelling capabilities were first assessed using 18F-labeled peptides injected in mice xenografted with a GMO, human AVPR2 positive cell line. This confirmed both the ability to for the first time selectively visualize ectopically expressed AVPR2 protein and the superiority of [18F]F-DBCO-MQ232. This radiotracer led to a total uptake of 3.5%ID.cc-3 in 600 mm^3^ tumours along with tumour/blood and tumour/muscle ratios of, respectively, 7 and 14. Those results are encouraging and bound to be further improved by a new radiolabeling approach. Murine and human ccRCC cell lines were then quantitatively screened for AVPR2 gene expression using RT-qPCR and for the presence of toxin-sensitive AVPR2 protein using an MQ-based fluorescent probe to develop a more clinic-like in vivo model. Results were compared with those of the positive control cell line and led to the development of a new model based on human Caki-1 cells. The efforts are now directed into the optimization of imaging protocol of this model as well as the development of a PDX mice model, the ultimate proof of concept.

**Keywords:** cancer; radiomarker; tumour

### 6.23. Cytotoxicity Profiling of Toxins in Snake Venoms Enabled by Nanofractionation Analytics and High Throughput Venomics

 Haifeng Xu ^1^, Matyas Bittenbinder ^1^, Julien Slagboom ^1^, Nicholas R. Casewell ^2^, Paul Jennings ^3^, Jeroen Kool ^1,^*
 ^1^ Amsterdam Institute of Molecular and Life Sciences, Division of BioAnalytical Chemistry, Vrije Universiteit Amsterdam, 1081 HV Amsterdam, The Netherlands.
 ^2^ Centre for Snakebite Research and Interventions, LSTM, Liverpool L3 5QA , United Kingdom.
 ^3^ Amsterdam Institute of Molecular and Life Sciences, Division of Molecular and Computational Toxicology, Vrije Universiteit Amsterdam, 1081 HV Amsterdam, The Netherlands.
 * Correspondence: j.kool@vu.nl

**Abstract:** Snake venom is well-known for causing severe hemotoxicity, neurotoxicity, and cytotoxicity upon envenoming. We evaluated four in vitro mammalian cell lines (RPTEC, HepaRG, HUVEC, and Hacat) against elapid venoms and their nanofractionated toxins for their cytotoxic properties. Fluorescence assays with Hoechst 33342 and propidium iodide, and the resazurin reduction assay were used to assess cell permeability and viability, respectively. For the toxins identified as cytotoxin, slight differences were observed in terms of the potency of cytotoxicity for the different cell lines investigated. Three crude cobra venoms (*Naja mossambica*, *N. haje*, and *N. naja*) showed dose-response cytotoxicity for all the cell lines by causing disruption of cell permeability and cell viability. *Bungarus multicinctus* venom only showed slight selective cytotoxicity in the HepaRG cell line. Susquencely, nanofractionation analytics coupled with parallel mass spectrometry was applied for separation and high-resolution fractionation of toxins with the acquirement of their accurate masses. Fractionated toxins were subjected to the four mammalian cellular assays for assessing the cytotoxicity profile of all individual toxins for all four cell lines, which are representative of the renal proximal tubule (RPTEC), liver (HepaRG), Human umbilical vein (HUVEC), and epidermis (Hacat) tissues. Next to this, a separate well plate with nanofractionated toxins was used for high-throughput venomics to chemically identify all fractionated toxins at the toxin ID level. PLA2s and 3FTXs were the prominent toxins found in nanofractionated venoms from *N. mossambica* and *N. naja* snakes that contributed to cytotoxicity. Furthermore, selective cytotoxicity was observed for some toxins based on the screening with the different cell lines. Through this, we developed and validated a cytotoxicity-assessing analytical platform that can be used to rapidly study cytotoxic venoms to pinpoint and identify their cytotoxins and obtain a clearer view of their cytotoxic mechanism of action, and selectivity towards different cell types.

**Keywords:** high-throughput venomics; in vitro cell assay; nanofractionation analytics; snake venom

### 6.24. Development of Nanofractionation Analytics for Identification of Venom Toxins Hitting Muscarinic Receptors

 Xiaoyi Zhang ^1,2,^*, Henry Vischer ^2^, Rob Leurs ^2^, Jeroen Kool ^1^
 ^1^ Division of Bioanalytical Chemistry, Amsterdam Institute of Molecular and Life Sciences, Vrije Universiteit, Amsterdam, 1081 HV Amsterdam, The Netherlands.
 ^2^ Division of Medicinal Chemistry Amsterdam Institute of Molecular and Life Sciences, Vrije Universiteit, Amsterdam, 1081 HV Amsterdam, The Netherlands.
 * Correspondence: x.zhang3@vu.nl

**Abstract:** Many peptide toxins in venoms have high target specificity and potency toward their prey receptors or ion channel targets. Some of these targets are also validated drug targets. Therefore, such venom toxins are considered good starting points for drug discovery and development in venoms-to-drugs pipelines. G protein-coupled receptors (GPCRs) play a dominant role in the regulation of human physiology in response to extracellular stimuli and are involved in many diseases. Consequently, GPCRs are one of the preferred drug target classes, with approximately 30% of the currently marketed drugs acting on a GPCR. So far, interesting toxins from several venoms have been identified as new GPCR ligands with exquisite selectivity and potency for the tested GPCR subtypes and have been developed into marketed drugs. Many others are underway in (pre-)clinical research. We aim to develop and apply an integrated workflow encompassing analytical chemistry and pharmacology, which can rapidly analyse large libraries of venoms for finding new GPCR ligands for various therapeutically relevant GPCRs. In this project, we plan to apply nanofractionation analytics to separate venoms by liquid chromatography followed by a flow split to parallel high resolution fractionation on well plates for subsequent parallel bioassay and mass spectrometry analysis to determine the accurate masses of bioactive toxins. In addition, high throughput (HT) venomics is planned to identify the toxins in the snake (*Dendroaspis polylepis* and *D. angusticeps*) venoms under study. GPCR bioactivity of fractionated toxins will be performed by dedicated radioligand binding assays. Next, all data measured are processed and presented as superimposed bioactivity, ultraviolet (UV), mass spectrometry (MS) and HT venomics protein score chromatograms. We will focus on muscarinic receptors as target GPCRs, which are involved in the parasympathetic nervous system. Finally, identified high-affinity muscarinic toxins from mamba venoms included in this study will be purified in larger quantity for further biological characterisation.

**Keywords:** bioactive toxin; muscarinic receptor; nanofractionation analytics; venom

## 7. Conclusions

**Figure 3:**
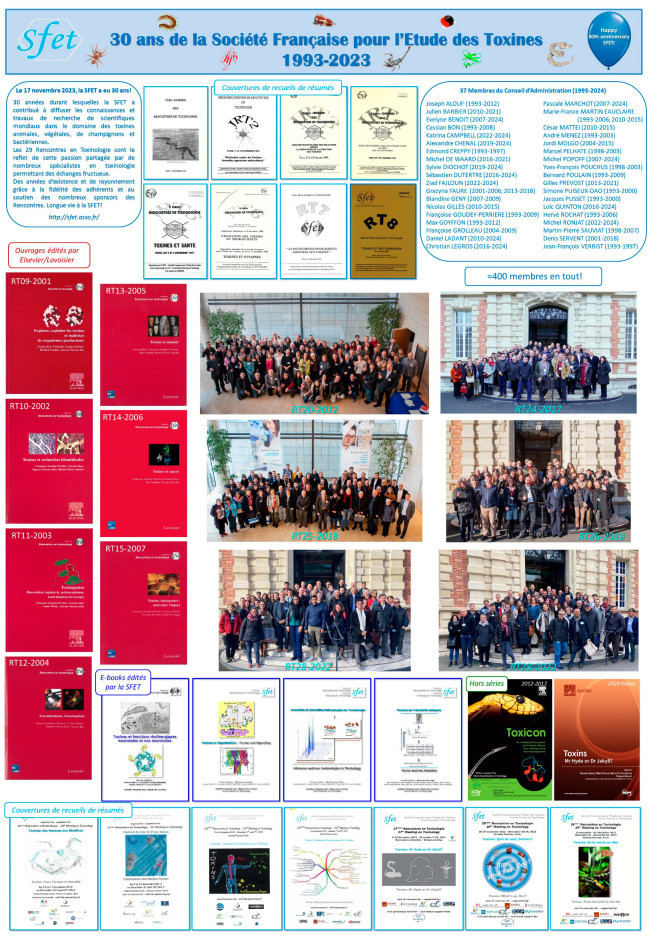
The 30th anniversary of the French Society on Toxinology (1993–2023).

The 29th Meeting on Toxinology of the SFET was another opportunity for fruitful exchanges on various toxinology themes, with prospects for extended collaboration between French, European and international research groups. We noted an increasing participation by researchers and students, with twice as many posters as during previous Meetings. Thanks to a grant from the MDPI journal Toxins, “Best Oral Presentation” and “Best Poster” awards were given to two young attendees by a panel of experts, after an animated discussion based on the high quality of all presentations. This Meeting also enabled us to commemorate the 30-year of existence of the SFET (Figure 3). We hope that the recent research on venoms and toxins, from their origin in a wide variety of animal, bacterial, fungal and plant species found in our environment to their exploration in the laboratory, will attract the submission of many manuscripts to this special issue.

## Data Availability

Not applicable.

